# Enzyme and lateral flow monoclonal antibody-based immunoassays to simultaneously determine spirotetramat and spirotetramat-enol in foodstuffs

**DOI:** 10.1038/s41598-021-81432-z

**Published:** 2021-01-19

**Authors:** Ramón E. Cevallos-Cedeño, Consuelo Agulló, Antonio Abad-Fuentes, Antonio Abad-Somovilla, Josep V. Mercader

**Affiliations:** 1 Institute of Agrochemistry and Food Technology (IATA), Spanish National Research Council (CSIC), Agustí Escardino 7, 46980 Paterna, Valencia Spain; 2 Department of Organic Chemistry, University of Valencia, Doctor Moliner 50, 46100 Burjassot, Valencia Spain; 3 Present Address: Department of Chemical Processes, Technical University of Manabi (UTM), Avenue José María Urbina y Che Guevara, 130105 Portoviejo, Ecuador

**Keywords:** Chemical safety, Bioanalytical chemistry, Immunological techniques, Biochemical assays, Small molecules

## Abstract

Spirotetramat is employed worldwide to fight insect pests due to its high efficiency. This chemical is quickly metabolized by plants into spirotetramat-enol, so current regulations establish that both compounds must be determined in foodstuffs for monitoring purposes. Nowadays, immunochemical methods constitute rapid and cost-effective strategies for chemical contaminant analysis at trace levels. However, high-affinity binders and suitable bioconjugates are required. In this study, haptens with opposite functionalisation sites were synthesized in order to generate high-affinity monoclonal antibodies. A direct competitive enzyme-linked immunosorbent assay with an IC_50_ value for the sum of spirotetramat and spirotetramat-enol of 0.1 μg/L was developed using selected antibodies and a novel heterologous bioconjugate carrying a rationally-designed hapten. Studies with fortified grape, grape juice, and wine samples showed good precision and accuracy values, with limits of quantification well below the maximum residue limits. Excellent correlation of results was observed with a standard reference chromatographic method. As a step forward, a lateral flow immunoassay was developed for onsite screening analysis of spirotetramat in wine. This assay was successfully validated according to Regulation 519/2014/EU for semi-quantitative methods at concentrations in line with the legal levels of spirotetramat and spirotetramat-enol in grapes, with a satisfactory false suspect rate below 2%.

## Introduction

**Figure 1:**
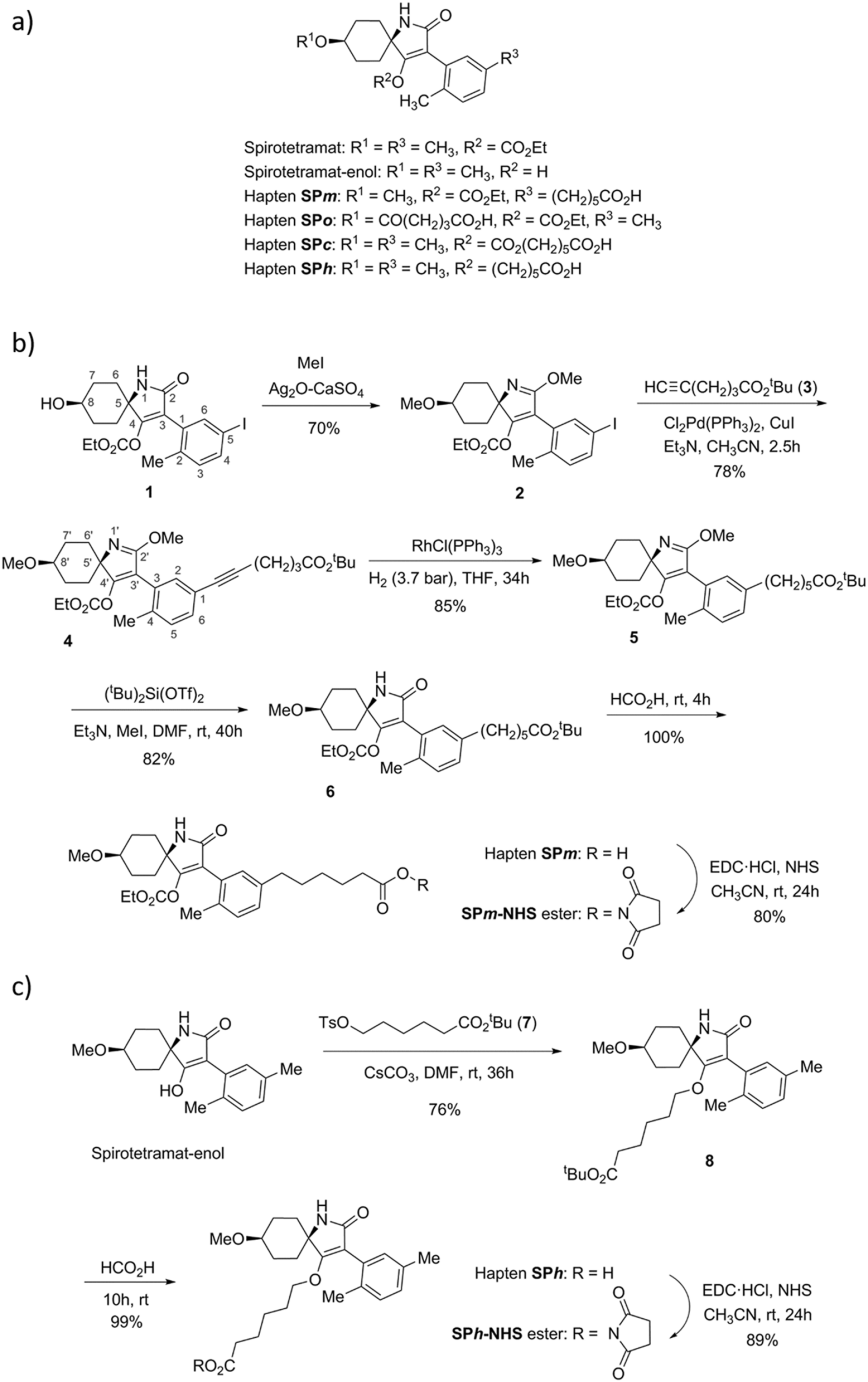
(**a**) Chemical structures of SP, SP-enol, and the four synthetic haptens. (**b**) Synthesis and activation of hapten SP*m*. (**c**) Synthesis and activation of hapten SP*h*.

Spirotetramat (SP), also known as BYI08330, was developed by Bayer CropScience a few years ago, and it was approved as a pesticide in the European Union and in the USA in 2014^1,2^. Nowadays, it is being commercialized worldwide as Movento, Ultor, or Kontos for insect pest control in a large variety of crops, such as stone fruits, pome fruits, berry fruits—including grapes—, citrus fruits, nuts, vegetables, etc. Originally derived from the natural antibiotic thiolactomycin, SP is structurally characterized by a cyclohexane ring spiro-linked tetramate core with three substituents: a methoxy, an ethoxycarbonyl, and a dimethylphenyl ring (Fig. 1a). This compound belongs to a new generation of active substances that show a novel highly efficient mode of action which blocks the biosynthesis of lipids by inhibiting the acetyl-CoA carboxylase activity in a broad spectrum of sucking insects in their juvenile stage, comprising psyllids, aphids, mealybugs, whiteflies, and scales^3,4^. After penetrating the leaf of the plant, SP is hydrolysed to spirotetramat-enol (SP-enol) so it can move along the phloem and the xylem, thus allowing to combat pests that are difficult to reach, such as the grape mealybug (*Planococcus ficus*)^5,6^. Further metabolism may occur in some crops, such as reduction or hydroxylation of the enol-carbonate double bond of SP-enol to form spirotetramat-monohydroxy (SP-mono) or spirotetramat-ketohydroxy (SP-keto), respectively. A fourth metabolite can occasionally be generated by glycosylation of the hydroxyl moiety, thus forming spirotetramat-enol-glucoside (SP-glu)^7^. SP does not show severe toxicity in mammals, though it may cause skin-sensitization and can be an eye irritant^8^. For those reasons, different residue definitions have been adopted by official regulatory agencies according to the target commodities and to the analytical purposes. In 2008, the Joint FAO/WHO Meeting for Pesticide Residues established the residue definition for monitoring the compliance with the maximum residue limits (MRL) for plant commodities as the sum of SP and SP-enol, expressed as SP^9^, and this definition was also adopted by the European Commission^10^.

Current analytical methods for SP residue determination in food commodities include high-performance liquid chromatography (HPLC) and gas chromatography (GC) coupled to mass spectrometry (MS) or other detection methods^11–13^. Additionally, the QuEChERS method (Quick, Easy, Cheap, Effective, Rugged, and Safe) has been set out as the most recommendable approach for residue extraction and sample preparation^14,15^. Zhu et al. optimized the analysis of SP residues by QuEChERS extraction and HPLC‒MS/MS in fruits and vegetables with limits of quantification (LOQ) in the low µg/kg range^16^. In 2015, Mohapatra et al. published the evaluation of SP and SP-enol in grapes by GC‒MS with a LOQ of 50 µg/kg^17^. Moreover, Berset et al. reported the determination of systemic insecticides, including SP, in commercial red and white wines by HPLC‒MS/MS with a LOQ of 1 µg/L^18^.

Nowadays, bioanalytical methods are being adopted as complementary screening tools for the rapid determination of chemical contaminants. Efficient sample preparation procedures and more robust and precise results have paved the way for the implementation of antibody-based methods, both in official analytical laboratories and in quality control departments of other stakeholders of the food chain. The high sensitivity, portability, and friendly use of immunochemical methods at competitive prices are being increasingly appreciated by the industrial sector. Recently, polyclonal antibodies to SP and SP-enol were generated showing the great potential of the studied synthetic strategy to prepare haptens^19^. In the present study, monoclonal antibodies (mAbs) to SP and SP-enol were raised for the first time with the aim to develop alternative immunoassays for simultaneous residue appraisal in food samples. In order to generate high-affinity mAbs with adequate specificity, a new immunizing hapten displaying the characteristic cyclohexane ring spiro-linked tetramate core was prepared. Moreover, an additional hapten was designed in order to obtain heterologous assay conjugates that could improve assay sensitivity and conjugate stability. Our goal was to optimize and validate different bioanalytical methods—a competitive enzyme-linked immunosorbent assay (cELISA) and a lateral flow immunochromatography assay—for total SP and SP-enol determination in relevant foodstuffs, such as grapes and their most commonly processed commodities, i.e., juice and wine.

## Results and discussion

### Hapten design and synthesis

Nobel prize-winner Karl Landsteiner demonstrated long ago the stereospecific binding behaviour of antibodies to haptens^20^. The nature and position of the chemical groups of the hapten are important for binding, but also the affinity and specificity of the newly-formed antibodies are greatly determined by the conformation of the molecule, and consequently the position of the spacer arm is of great relevance^21^. Nevertheless, the optimum linker tethering site is difficult to predict, particularly when antibodies with broad specificity are searched. Taking into account these premises, haptens were designed to generate high-affinity antibodies to SP and its metabolite SP-enol, and to develop highly sensitive immunoassays (Fig. 1a). In a previous study, the bovine serum albumin (BSA) conjugate of hapten SP*o*—with the spacer arm at the aliphatic ring, distal to the aromatic ring of the SP molecule—afforded good polyclonal antibodies^19^, so it was used in the present study to generate monoclonals. In that same study, the conjugate BSA‒SP*c*—with the linker in a central position of the hapten molecule—did not render good antibodies, most probably because of the lability of the carbonate moiety, so it was discarded for immunization of mice. Now, a novel immunizing hapten (SP*m*) with the spacer arm at the aromatic ring of SP, which is opposite and complementary to that of hapten SP*o*, was designed. Additionally, with a view to developing immunoanalytical methods, and because hapten heterology has often been demonstrated as a good strategy to improve the performance of immunoassays, hapten SP*h*—a more stable variant of hapten SP*c* in which the labile carbonate group of the spacer arm was replaced by an ether bond—was conceived.

Hapten SP*m* was prepared by total synthesis from aryl iodide **1**, which in turn was obtained, as detailed in the Supplementary Information file (Figure S1), from 1,4-cyclohexanedione monoethylene acetal and 2-(*o*-tolyl) acetic acid. This procedure followed a synthetic route similar to that previously used for the preparation of the methylated analogue at the C-5 position of the phenyl ring^19,22^. First, *O*-methylation of the hydroxyl group at the C-8 position of the 1-azabicyclic moiety was carried out (Fig. 1b). Attempts to methylate this position using methyl iodide as methylating agent always led to partial *O*-methylation of the amide group, so we decided to perform an exhaustive methylation of both positions for later—once the incorporation of the spacer arm was completed—regenerate the amide group. The *O*-methylation of both the hydroxyl and amide groups was undertaken in good yield by treatment of **1** with an excess of MeI in the presence of silver oxide and drierite (anhydrous calcium sulphate). The incorporation of the carboxylated spacer arm as its *tert*-butyl ester was based on the Sonogashira cross-coupling reaction between the iodinate position of **2** and the terminal alkyne-ester **3**, which was previously prepared from commercially available hex-5-ynoic acid^23^. The cross-coupling reaction was carried out efficiently using Cl_2_Pd(PPh_3_)_2_ as the source of the palladium catalyst, CuI as co-catalyst, and Et_3_N as the base, in DMF at room temperature. The incorporation of the carboxylated aliphatic linear chain was completed from the alkyne resulting from the cross-coupling reaction, i.e. **4**, by hydrogenation reaction of the triple bond under homogeneous conditions using Wilkinson's catalyst. The synthesis of hapten SP*m* was readily completed from intermediate **5** by cleavage of both the methoxy group—to regenerate the unsubstituted amide group—and the *tert*-butyl ester group—to form the corresponding carboxylic moiety. Thus, demethylation of the methoxy-2H-pyrrole ring of **5**, followed by acid catalysed cleavage of the *tert*-butyl ester group, afforded the target hapten SP*m* in an excellent global yield. Overall, the synthesis of hapten SP*m* from aryl iodide **1** proceeded in 5 steps with a total yield of *ca*. 40%.

The synthesis of the heterologous hapten SP*h*, which incorporates the spacer arm through a C‒O ether linkage at the enolic oxygen atom, was significantly simpler than that of hapten SP*m* (Fig. 1c). The synthesis starts from SP-enol, obtained by alkaline hydrolysis of SP, and involves the *O*-alkylation reaction of the enolic hydroxyl group with the primary tosylate **7**^24,25^, using caesium carbonate as the basic catalyst, to give the enol ether **8** in good yield. As in the synthesis of hapten SP*m*, the preparation of hapten SP*h* was finished by acid catalysed cleavage of the *tert*-butyl ester moiety of **8**, a reaction that, as in the previous case, also occurred with a practically quantitative yield. The two-step sequence provided hapten SP*h* in 75% overall yield from SP-enol.

### Hapten activation and bioconjugate preparation

Previously to conjugation to the carrier proteins, haptens SP*m* and SP*h* were activated through the formation of the corresponding active esters. The synthesis of the *N*-hydroxysuccinimidyl ester of hapten SP*m* was initially carried out using *N,N*-disuccinimidyl carbonate and Et_3_N as the catalyst, conditions that led to obtaining the corresponding active ester with relatively low yields (< 50%). A much higher yield was found under non-basic conditions using 1-ethyl-3-(3-dimethylaminopropyl)carbodiimide hydrochloride (EDC·HCl) and *N*-hydroxysuccinimide (NHS) in dry acetonitrile at room temperature for 24 h. Under these conditions, the *N*-hydroxysuccinimidyl ester SP*m*-NHS was obtained in 80% yield (Fig. 1b). An even higher yield (89%) was attained when the same conditions were used for the preparation of the analogue ester of hapten SP*h* (Fig. 1c).

BSA, ovalbumin (OVA), and horseradish peroxidase (HRP) were employed as carrier proteins to prepare bioconjugates with the corresponding active esters of haptens SP*m* and SP*h*. The obtained protein conjugates were purified by size exclusion chromatography and the hapten density was determined by MALDI-TOF‒MS. Hapten-to-protein molar ratios of *ca*. 12.9, 3.8, and 0.5 for BSA–SP*m*, OVA–SP*m*, and HRP–SP*m* conjugates, and 17.9, 10.5, and 1.2 for BSA–SP*h*, OVA–SP*h*, and HRP–SP*h* conjugates, respectively, were obtained, which are equivalent to those of the previously published SP*o* conjugates^19^. MALDI-TOF mass spectra (Figures S2‒S4) of the newly prepared bioconjugates are provided in the Supplementary Information file.

### Antibody generation and characterization

Six mouse mAbs were obtained; three were from immunogen BSA‒SP*m*—named SP*m*#23, SP*m*#25, and SP*m*#216—and three from conjugate BSA‒SP*o*—named SP*o*#227, SP*o*#237, and SP*o*#243. These antibodies contained κ light chains and were of the IgG_1_ isotype, except mAb SP*m*#25 which was IgG_2a_. The affinity to SP and SP-enol was studied by checkerboard cELISA in the capture antibody-coated direct format, using homologous and heterologous enzyme tracers (with the same or different hapten, respectively, than the immunizing conjugate). SP and SP-enol standards were prepared in PBS (10 mM phosphate buffer, pH 7.4, with 140 mM NaCl) and tracer solutions for the competitive step were prepared in PBS-T (PBS containing 0.05% (v/v) Tween-20). Under these conditions, A_max_ values higher than 0.8 were achieved with four antibodies (SP*m*#23, SP*m*#216, SP*o*#237, and SP*o*#243) combined with the homologous enzyme conjugate (Table 1). Tracers with linker site heterologies were not bound by the antibodies. The only exception was mAb SP*m*#23 which did recognize the heterologous HRP‒SP*h* conjugate. For this antibody, the spacer arm of hapten SP*h* was located at a more proximal site than that of hapten SP*o*. The lowest IC_50_ value with the homologous tracer was around 3 nM for SP-enol whereas binding to SP was moderate (IC_50_ values were higher than 30 nM). Interestingly, a very low IC_50_ value (0.55 nM) for SP-enol was obtained with mAb SP*m*#23 combined with HRP‒SP*h* heterologous tracer.

**Table 1:** Checkerboard assay using the capture antibody-coated direct cELISA (n = 3).

	Tracer conjugate														
	HRP‒SP*m*					HRP‒SP*o*					HRP‒SP*h*				
mAb	[mAb]	[T]	A_max_	IC_50_ SP	IC_50_ SPenol	[mAb]	[T]	A_max_	IC_50_ SP	IC_50_ SPenol	[mAb]	[T]	A_max_	IC_50_ SP	IC_50_ SPenol
SP*m*#23	100	30	1.03	33.7	2.83	1000	300	–			100	30	1.25	8.50	0.55
SP*m*#25	1000	300	–			1000	300	–			1000	300	–		
SP*m*#216	1000	300	0.92	324	10.8	1000	300	–			1000	300	–		
SP*o*#227	1000	300	–			1000	300	–			1000	300	–		
SP*o*#237	1000	300	–			100	30	1.53	44.1	8.56	1000	300	–		
SP*o*#243	1000	300	–			1000	300	1.15	249	8.08	1000	300	–		

[mAb], antibody concentration in μg/L; [T], tracer concentration in μg/L; IC_50_, values are expressed in nM units; –, signal was lower than 0.8.

Concerning specificity, the two assayed immunogens (BSA‒SP*m* and BSA‒SP*o*) afforded equivalent results. Binding to both SP and SP-enol was observed with the two types of antibodies, and SP-enol was always better recognized. The partial in vivo hydrolysis of the carbonate group of haptens SP*m* and SP*o* during immunization could explain this result. Cross-reactivity with the main SP metabolites was studied with antibody SP*m*#23. The affinity of this mAb to SP-enol was at least 10 and 20 times higher than to SP and SP-glu, respectively—binding to SP-keto and SP-mono was almost negligible (Table S1 in the Supplementary Information file). Moreover, these antibodies did not bind to other structurally related pesticides, such as spiromesifen, spiroxamine, and spirodiclofen, neither to other insecticides that could be present in food samples, such as imidacloprid and deltamethrin, nor to fungicides that are commonly used in fruit crops, such as dimoxystrobin, azoxystrobin, pyraclostrobin, procymidone, fenpropimorph, fenamidone, boscalid, propamocarb, cyprodinil, pyrimethanil, fludioxonil, fenhexamid, thiabendazole, *o*-phenylphenol, chlorothalonil, and mandipropamid.

### Competitive ELISA development

Antibody SP*m*#23 combined with tracer HRP‒SP*h* were selected for cELISA development in the capture antibody-coated direct format. To begin with, the influence of pH and ionic strength over the main parameters of the standard curve of SP-enol was evaluated. Standards were prepared in MilliQ water and tracer solutions for the competitive step were prepared in 20 mM phosphate buffer, at the studied pH and NaCl concentrations, and containing 0.05% (v/v) Tween-20. It was observed that the studied immunoassay was quite tolerant to pH changes between pH 6.0 and 8.5 (Figure S5 in the Supplementary Information file). On the contrary, the immunoassay was sensitive to ionic strength changes—low salt concentrations strongly diminished the A_max_ value. However, higher salt concentrations increased the A_max_ value, though little effects on the IC_50_ value were observed. Tolerance to ethanol and acetonitrile was also assessed with the selected direct cELISA because the former solvent will be present in one of the studied samples (wine) and the latter will be used to extract solid samples (grapes). Standards were prepared in the studied solvent dilutions in MilliQ water, and tracer solutions for the competitive step were prepared in 20 mM phosphate buffer, pH 7.4, containing 280 mM NaCl and 0.05% (v/v) Tween-20. It was observed that none of the evaluated solvents was well tolerated by the selected immunoassay (Figure S6 in the Supplementary Information file). Contents higher than 1% (v/v) were detrimental; particularly, the A_max_ value rapidly decreased with increasing solvent concentrations. The IC_50_ value considerably increased with the presence of ethanol whereas it was not so much influenced by acetonitrile.

It is known that SP quickly hydrolyses to form SP-enol at basic pH values^4^. In order to simultaneously quantify SP and SP-enol contents in a sample, an in situ procedure for treating the sample with alkaline solution in order to hydrolyse SP into SP-enol was assessed. SP and SP-enol standard solutions were prepared in MilliQ water and diluted fivefold with 40 mM NaOH. The mixtures were incubated 10 min at room temperature and diluted twofold with dilution buffer (200 mM Tris·HCl buffer, pH 8.0, containing 280 mM NaCl). For the competitive step, enzyme tracer solutions were prepared in TBS-T (100 mM Tris·HCl buffer, pH 8.0, containing 140 mM NaCl and 0.05% (v/v) Tween-20). As shown in the inset of Table 2, the obtained standard curves of SP-enol and hydrolysed SP were almost identical. This hydrolysis procedure was validated by HPLC‒MS/MS. SP and SP-enol aqueous solutions (50 µg/mL) were treated with NaOH and, 10 min later, the pH was lowered as previously described. Then, 10 µL of hydrolysed and diluted sample was transferred into 990 µL of acetonitrile and dried with anhydrous MgSO_4_. Controls without the hydrolysis step were also evaluated. Finally, the contents of SP, SP-enol, SP-keto, and SP-mono in the organic extracts were quantified (Table 2). We observed that when SP was incubated in a basic solution (sample A), it was completely hydrolysed into SP-enol, and none of the other metabolites was formed. On the contrary, SP-enol remained unmodified even if it was treated with NaOH (sample C). Recovery values between 88.8% and 119.6% were obtained. Those samples were also analysed with the studied direct cELISA by running SP-enol standard solutions that had been incubated in NaOH and diluted with dilution buffer like the samples. The tracer solution for the competitive step was prepared in TBS-T. When SP and SP-enol were incubated in NaOH solution (samples A and C), the recovery values—measured as SP-enol—were 85.0% and 117.6%, respectively. As expected, the non-hydrolysed SP control solution (sample B) could not be quantified, whereas the recovery was 118.0% when the SP-enol control solution (sample D) was analysed. Consequently, the developed hydrolysis procedure seemed to perform adequately for SP and SP-enol residue quantification as SP-enol equivalents.

**Table 2:**
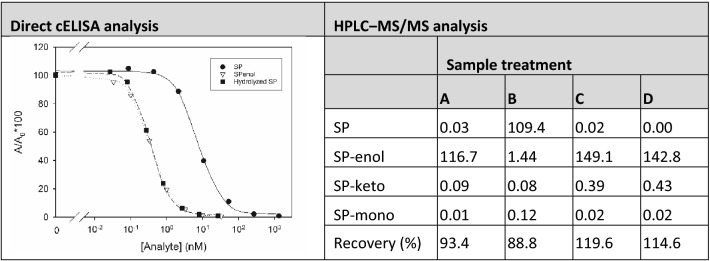
Spirotetramat hydrolysis study by direct cELISA and HPLC‒MS/MS.

A, SP hydrolysed; B, SP without hydrolysis; C, SP-enol hydrolysed; D, SP-enol without hydrolysis.

The sigmoidal inhibition curve obtained for SP-enol and the final assay conditions of the developed direct cELISA are provided in Table 3. High sensitivity for SP-enol was found—the IC_50_ value was 0.34 nM (0.1 μg/L)—under the optimized conditions. The A_max_ value of the standard curve was near 1.0, the slope was − 1.4, and the background signal was negligible. The calculated limit of detection (LOD) value was 0.02 μg/L, and the dynamic working range was between 0.05 and 0.31 μg/L. Moreover, excellent intra-day and inter-day precision values (below 10%) for the A_max_ and IC_50_ values were observed.

**Table 3:** Assay conditions and analytical parameters of the optimized immunoassay (n = 5).

Standard curve and assay parameters	

mAb	SP*m*#23 100 μg/L
Conjugate	HRP–SP*h* 30 μg/L
Assay buffer	100 mM Tris·HCl, pH 8.0, 140 mM NaCl, 0.05% Tween-20
Hydrolysis solution	40 mM NaOH
Dilution buffer	200 mM Tris·HCl, pH 8.0, 280 mM NaCl
A_max_	0.901 ± 0.044
IC_50_ SP-enol (nM)	0.344 ± 0.025
IC_50_ SP-enol (μg/L)	0.104 ± 0.007
Slope	− 1.430 ± 0.112
A_min_	0.001 ± 0.003
LOD (μg/L) (IC_10_)	0.022 ± 0.004
Dynamic range (μg/L)	
(IC_20_‒IC_90_)	0.048‒0.318
Inter-day precision	
A_max_ (%)	4.7
IC_50_ (%)	7.9
Intra-day precision	
A_max_ (%)	2.9
IC_50_ (%)	9.7

### Competitive ELISA validation

The performance of the developed immunoassay to analyse SP and SP-enol in wine, grape juice, and grape extracts was assessed. Solid samples were extracted by the QuEChERS method. To study the matrix effects, SP-enol standard solutions were prepared in wine, juice samples, and organic extracts, and each solution was diluted to a different extent with 40 mM NaOH. After 10 min, samples were 1:1 diluted with dilution buffer, and wine and juice samples were cleaned-up with polyvinylpolypyrrolidone (PVPP). The enzyme tracer solution was prepared in TBS-T. The matrix effects of wine, grape juice, and grape extracts over the A_max_ and IC_50_ values are depicted in Figure S7 in the Supplementary Information file. We observed that the deviation of the A_max_ value was mostly acceptable even at a low dilution of the samples, except for the red wine, and the deviation of the IC_50_ value was acceptable or just slightly below − 20% at a fivefold dilution factor for all of the assayed food samples.

SP-free samples were fortified between 2.5 and 500 μg/L with SP, SP-enol, and a 1:1 mixture of both compounds. Samples were diluted with 40 mM NaOH solution and incubated, and then 1 volume of dilution buffer was added. Wine and juice samples were cleaned-up with PVPP. Recovery values were determined from a SP-enol standard curve run in the same microtiter plate. Excellent recovery values between 80 and 120%, and coefficients of variation below 20% were obtained with the developed direct cELISA (Table 4) for the five studied matrices, independently of the analyte and even with mixtures of both compounds. Thus, the LOQ for SP and SP-enol of this immunoassay can be set at 2.5 μg/L for wine, grape juice, and grapes.

**Table 4:** Recovery values (as SP-enol) and coefficients of variation obtained with fortified samples by the developed direct cELISA (n = 3).

Sample	[A]	SP			SP-enol			SP + SP-enol (1:1)		
		d.f	R (%)	CV (%)	d.f	R (%)	CV (%)	d.f	R (%)	CV (%)
Grapes	2.5	25	–	–	25	–	–	25	–	–
	5.0	25	113.9	10.3	25	121.7	8.3	25	121.3	7.3
	10	25	99.7	9.3	25	113.4	12.0	25	113.9	18.3
	25	25	107.2	12.8	25	112.9	5.5	25	116.5	14.2
	50	50	92.7	6.6	50	99.9	7.1	50	94.2	17.1
	100	50	98.1	10.9	50	99.6	8.3	50	98.8	18.9
	250	50	85.6	8.5	50	98.3	7.1	50	91.2	13.9
	500	50	83.7	1.7	50	96.2	9.6	50	91.9	14.6
White grape juice	2.5	5	88.4	2.7	5	116.5	4.8	5	81.5	6.3
	5.0	5	90.6	4.5	5	113.6	6.8	5	84.9	2.2
	10	5	98.5	13.4	5	114.0	5.8	5	88.7	10.8
	25	5	99.9	11.5	5	112.5	10.3	5	88.5	17.3
	50	50	102.0	6.5	50	106.4	5.2	50	83.7	3.1
	100	50	115.3	11.3	50	109.3	8.6	50	89.4	9.1
	250	50	102.8	10.8	50	97.0	7.5	50	83.8	13.9
	500	50	85.4	8.4	50	97.3	10.8	50	89.8	6.8
Red grape juice	2.5	5	98.4	5.2	5	112.9	1.7	5	92.3	15.3
	5.0	5	92.0	4.3	5	115.2	2.7	5	88.5	15.6
	10	5	101.5	8.0	5	115.5	3.7	5	87.8	6.5
	25	5	103.0	4.7	5	118.0	1.3	5	82.8	7.1
	50	50	109.9	9.8	50	106.6	2.2	50	88.5	7.2
	100	50	109.6	3.4	50	105.5	3.5	50	89.7	2.8
	250	50	103.7	3.0	50	103.3	1.4	50	82.8	8.7
	500	50	86.1	3.0	50	115.0	3.7	50	85.4	2.0
White wine	2.5	5	115.4	2.9	5	113.2	10.3	5	115.9	5.6
	5.0	5	106.8	5.9	5	113.9	11.3	5	104.3	16.6
	10	5	106.6	2.2	5	115.3	14.1	5	89.6	13.4
	25	5	100.8	9.2	5	105.4	8.5	5	82.9	4.5
	50	50	93.2	8.8	50	104.5	10.9	50	95.0	2.5
	100	50	102.1	4.3	50	103.4	6.1	50	88.3	7.5
	250	50	93.3	3.8	50	104.7	2.0	50	81.1	6.4
	500	50	86.2	10.3	50	91.1	1.2	50	81.7	9.0
Red wine	2.5	5	108.3	10.1	5	107.8	14.7	5	123.4	5.9
	5.0	5	97.6	11.4	5	107.2	15.4	5	117.9	16.8
	10	5	96.9	14.2	5	113.3	18.6	5	103.1	8.7
	25	5	93.0	10.8	5	109.7	5.6	5	92.6	8.7
	50	50	89.6	14.2	50	96.9	14.2	50	101.0	10.9
	100	50	91.7	10.5	50	104.3	10.5	50	98.5	9.9
	250	50	88.2	11.3	50	107.3	18.1	50	94.0	10.8
	500	50	88.1	2.3	50	98.4	1.3	50	94.5	20.0

[A], analyte concentration in μg/L; d.f., sample dilution factor in NaOH; R, recovery values. –, out of range.

**Figure 2:**
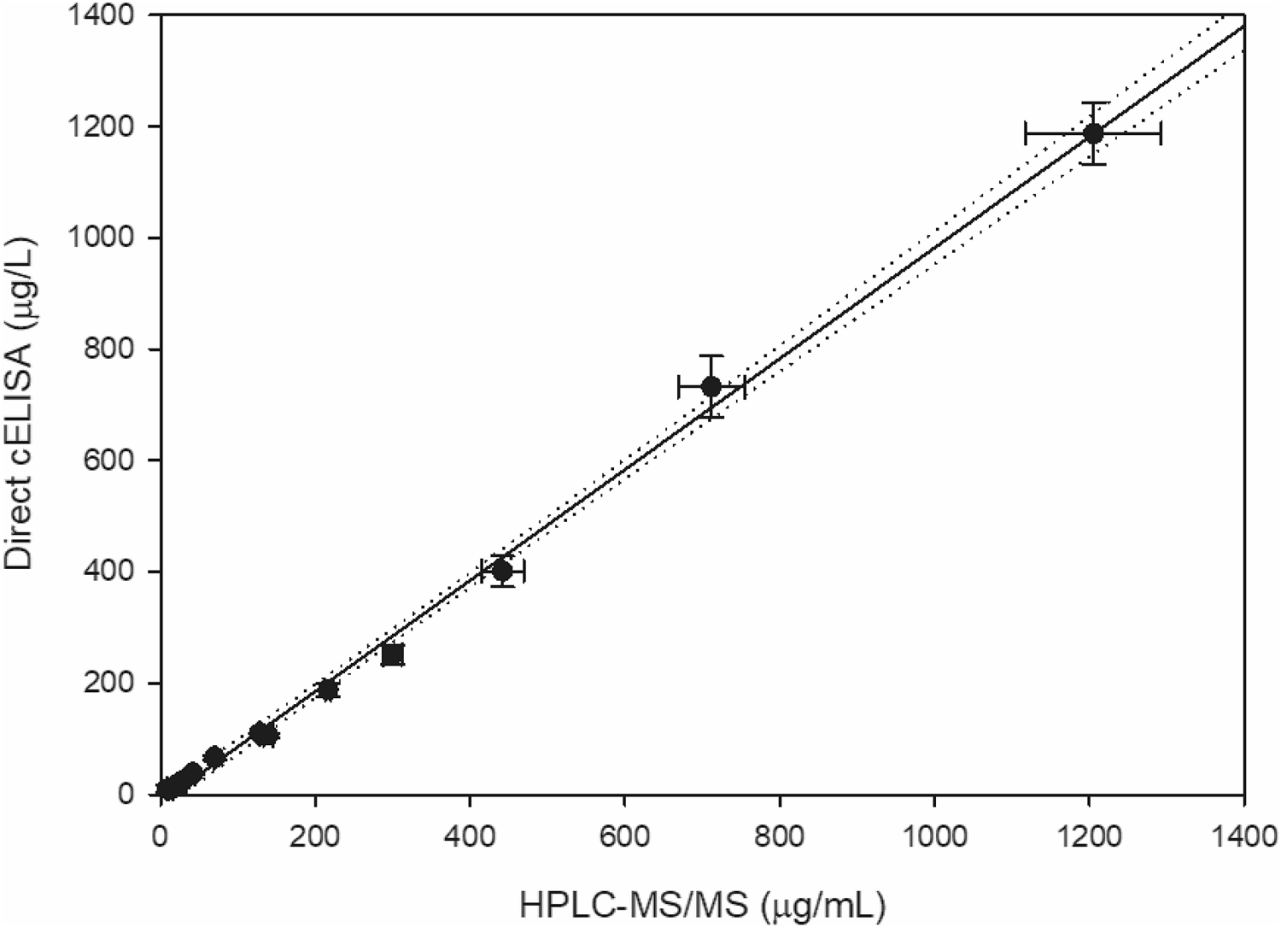
Comparison of results obtained by direct cELISA and HPLC‒MS/MS from contaminated grape samples.

To further validate the direct cELISA for SP residue analysis in food samples, analytical results were compared with a reference chromatographic method. Grapes were sprayed once or twice with a solution of Movento Gold containing 10, 80, or 160 mg/L of SP in tap water; then, berries were homogenized and extracted by the QuEChERS procedure and the extracts were analysed by both analytical methods. The obtained results are listed in Table S2. The bias between both sets of results was mainly between 80 and 120%. Figure 2 depicts the regression line and the corresponding 95% confidence interval (CI) for comparison of results. The intercept was − 13.051 and the CI was from − 28.400 to 2.299, so it was statistically comparable to zero. The slope of the regression line was 0.995, and the CI for this parameter was 0.959‒1.031, so it included the 1.0 value. Therefore, the developed direct cELISA and the reference chromatographic method afforded equivalent results.

### Lateral flow immunoassay

A lateral flow immunochromatography assay was optimized and characterized using BSA‒SP*h* for the test line, GAM (goat anti-mouse immunoglobulins polyclonal antibody) for the control line, and gold nanoparticle (GNP)-labelled antibody SP*m*#23 in the mobile phase. To optimise the amount of GNP conjugate as well as the pH and ionic strength of the assay buffer, tests were run with different standard concentrations and a blank. SP-enol standard solutions were 0.1 and 0.3 μg/L and SP standard solutions were 0.3 and 1.0 μg/L. The immunoassay response was determined as the quotient between the signal of the test line (T) and the signal of the control line (C), and the inhibition rate was calculated. Figure S8 shows the T/C ratios and inhibition rates obtained with varying amounts of GNP‒mAb conjugate. The highest inhibition rate was obtained with the lowest assayed quantity of bioconjugate (5 µL); however, the T and C values were too low for reading with the naked eye. Therefore, 10 µL of GNP‒mAb conjugate was chosen as the optimum volume for a total of 100 µL of reaction mixture. Additionally, the pH and the ionic strength were optimized for this lateral flow immunoassay (Figures S9 and S10). The inhibition rate was not significantly changed at pH 7.5, 8.0, and 8.5, whereas a somewhat higher inhibition rate was found at 60 mM NaCl concentration. Consequently, 100 mM Tris·HCl buffer, pH 8.0, containing 60 mM NaCl and 0.05% (v/v) Tween-20 was chosen as assay buffer for immunochromatographic analysis.

**Figure 3:**
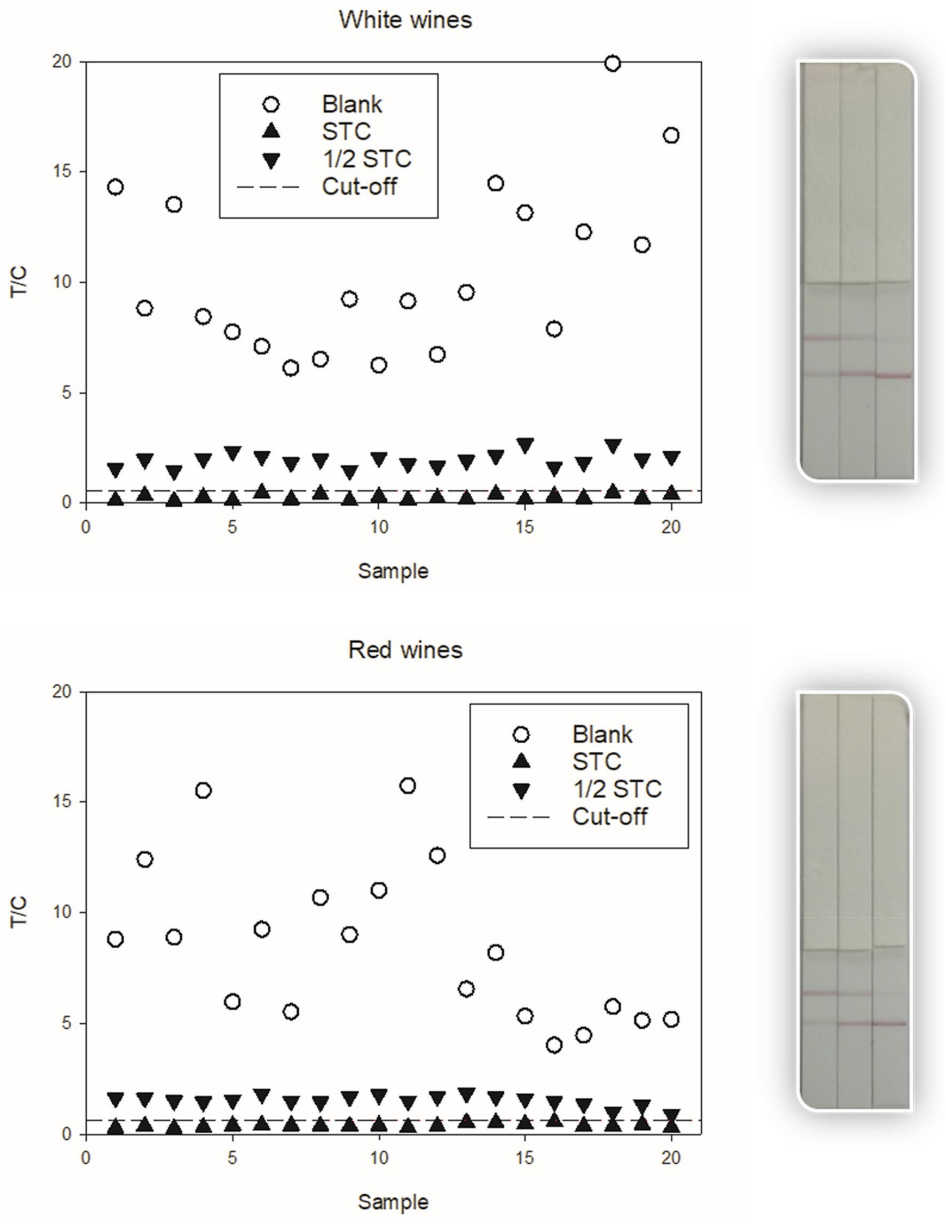
Lateral flow immunoassay response and cut-off values for SP determination in wine samples obtained for blank samples and samples containing SP at STC and 50% STC. The immunostrips show the obtained results for wine samples fortified with SP at (from left to right) 1000, 500, and 0 μg/L, and treated as optimized to hydrolyse SP into SP-enol.

This rapid test was applied to the semi-quantitative determination of SP and SP-enol residues in wine samples. The cut-off values for white and red wines—the reference T/C values to discriminate between negative and suspect samples—and the rates of false suspect results of the optimized lateral flow immunoassay were determined for a 95% CI^26,27^. The 2014 EU Regulation 519/2014/EU for validation of semi-quantitative screening analytical methods with inversely proportional response was followed^28^. Two white wine and two red wine samples were fortified with SP or SP-enol and fivefold diluted in 40 mM NaOH solution. After 10 min incubation at room temperature, they were 100-fold diluted with assay buffer. Each sample was measured twice every day during 5 consecutive days (n = 20). The European MRL for SP in wine grapes is 2000 μg/kg, but no MRL has been set so far for wines. Nevertheless, an average transfer factor from grapes to wine of 0.5 has been previously reported by EFSA (European Food Safety Authority)^10^. Consequently, the screening target concentration (STC) for SP in this study was set at 1000 μg/L. Figure 3 shows the obtained T/C values for the blank and the fortified samples with SP at a concentration equal to the STC and to 1/2 STC. Equivalent results were obtained for samples fortified with SP-enol (Figure S11 and Table S3). For 19 degrees of freedom and a *t*-value of 1.7291 for a 5% ratio of false negative results at the STC, the obtained cut-off values were 0.5 for the two types of wines. Moreover, the rates of false suspect results—blank samples that could be classified as suspect—were below 2% for both wines (Table 5). The rates of false suspect results for samples containing SP residues at 500 μg/L (1/2 STC) were almost negligible for both types of wine. Finally, the probability for a wine sample containing 1000 μg/L of SP to be classified as contaminated but containing a residue concentration equivalent to 1/2 of STC—false negative from STC—was null. The visual LOD (vLOD) for SP residue monitoring in wine by the developed lateral flow immunoassay could be set at 1000 μg/L, as seen in Fig. 3. Therefore, the optimized procedure fits the European requirements for the semi-quantitative rapid analysis of SP as SP-enol in wine by the developed lateral flow immunoassay.

**Table 5:** Validation of the lateral flow immunoassay to determine SP residues as the sum of SP + SP-enol in wine samples (n = 20).

	White wines		Red wines	
	STC	1/2 STC	STC	1/2 STC
Average T/C	0.3	2.0	0.4	1.5
CV (%)	45.7	17.0	22.0	16.0
Cut-off (5% negative results)	0.5	2.8	0.5	1.9
False suspect (%) from blank	1.7	3.1	1.9	4.1
False suspect (%) from 1/2 STC	0.01	–	0.03	–
False negative (%) from STC	–	0.00	–	0.00

The STC was set at 1000 μg/L.

## Conclusions

High-affinity mAbs with similar specificity were obtained both from hapten SP*o*, which displays the SP aromatic ring from a distal site, and from hapten SP*m*, which has an aliphatic ring opposite to the linker tethering site. This finding has been also observed with haptens for a different small chemical compound^29^. Hapten SP*h* was an adequate approach to improve immunoassay sensitivity. An innovative strategy was optimised to quickly and quantitatively transform SP into SP-enol. With this sample preparation procedure, the developed direct cELISA showed an excellent performance to precisely measure SP residues as the sum of SP + SP-enol in grape extracts, grape juice and wine, with LOQ values well below the European MRL for grapes. Finally, the optimized immunochromatographic assay performs perfectly for the screening of total SP residues in wine at the legal levels.

## Methods

### Reagents and instruments

Chemicals and apparatus, as well as general experimental techniques that were employed for the synthesis of novel haptens, for the preparation of bioconjugates, for hybridoma generation, and for immunoassay development are listed and described in the Supplementary Information file. SP (ethyl cis-8-methoxy-2-oxo-3-(2,5-xylyl)-1-azaspiro[4.5]dec-3-en-4-yl carbonate; CAS registry number 203313–25-1; MW 373.45), SP-enol, SP-keto, SP-mono, and SP-glu analytical standards, Pestanal grade, were purchased from Merck. Pesticide stock solutions were prepared in amber glass vials using *N*,*N*-dimethylformamide (DMF) as solvent, and kept at − 20 °C. Technical SP and formulated SP (Movento Gold 10% SC) were kindly provided by Bayer CropScience.

### Hapten synthesis

All reactions involving air-sensitive compounds were conducted in oven-dried glassware under a nitrogen atmosphere. The synthesis of hapten SP*o* was reported in a previous study^19^. Details of all of the synthetic steps and characterization data of intermediate compounds are provided in the Supplementary Information file.

Hapten SP*m* was obtained in five synthetic steps as a semisolid (Fig. 1b). IR ν_max_ (cm^−1^) 3400‒2600 (m), 3220 (m), 2938 (s), 2859 (m), 1779 (s), 1714 (s), 1214 (s), 1100 (m), 756 (m), 734 (m); ^1^H NMR (CDCl_3_, 300 MHz) δ 7.83 (1H, s, NH), 7.12 (1H, d, *J* = 7.8 Hz, H-5 Ph), 7.03 (1H, dd, *J* = 7.8, 1.7 Hz, H-6 Ph), 6.95 (1H d, *J* = 1.7 Hz, H-2 Ph), 3.99 (2H, q, *J* = 7.1 Hz, CH_3_*CH*_*2*_OCO_2_), 3.38 (3H, s, C8′-OCH_3_), 3.23 (1H, dq, *J* = 10.1, 5.2, 4.0 Hz, H-8′), 2.55 (1H, t, *J* = 7.6 Hz, H-6), 2.31 (2H, t, *J* = 7.2 Hz, H-2), 2.26‒2.13 (2H, m, H-7′/H-9′), 2.22 (3H, s, CH_3_-Ph), 1.92 (2H, td, *J* = 13.6, 3.5 Hz, H-6′/H-10′), 1.81‒1.71 (2H, m, H′-6′/H′-10′), 1.71‒1.53 (4H, tt, *J* = 16.0, 7.8 Hz, H-3 and H-5), 1.50‒1.42 (2H, m, H′-7′/H′-9′), 1.42‒1.31 (2H, m, H-4), 1.09 (3H, t, *J* = 7.1 Hz, *CH*_*3*_CH_2_OCO_2_); ^13^C NMR (CDCl_3_, 75 MHz) δ 178.5 (C-1), 171.3 (NCO), 165.4 (C-4′), 150.0 (OCO_2_), 139.8 (C-1 Ph), 134.5 (C-3 Ph), 130.4 (C-5 Ph), 129.5 (C-2 Ph), 129.0 (C-6 Ph), 127.8 (C-4 Ph), 121.6 (C-3′), 77.5 (C-8′), 65.9 (CH_3_*CH*_*2*_OCO_2_), 60.9 (C-5′), 55.9 (OCH_3_), 35.2 (C-6), 34.3 (C-2), 31.7 (C-6′/C-10′), 31.1 (C-5), 28.7 (C-4), 28.5 (C-7′/C-9′), 24.8 (C-3), 19.4 (CH_3_-Ph), 13.9. (CH_*3*_CH_2_OCO_2_); HRMS (TOF–MS ES+) calcd for C_26_H_36_NO_7_ [M + H]˙^+^ 474.2486, found 474.2484.

Hapten SP*h* was obtained from SP-enol in two synthetic steps (Fig. 1c). IR ν_max_ (cm^−1^) 3265 (broad, w), 2942 (m), 2868 (w), 1702 (s), 1685 (s), 1648 (s), 1337 (s), 1104 (s), 935 (w), 813 (w); ^1^H NMR (CDCl_3_, 300 MHz) δ 7.69 (1H, s, NH), 7.07 (1H, d, *J* = 7.7 Hz, H-3 Ph), 7.01 (1H, dd, *J* = 7.6, 1.4 Hz, H-4 Ph), 7.00 (1H, br s, H-6 Ph), 3.73 and 3.62 (1H each, each dt, *J* = 10.1, 6.2 Hz, H-6 and H′-6), 3.38 (3H, s, OCH_3_), 3.24 (1H, tt, *J* = 10.8, 4.0 Hz, H-8′), 2.29 and 2.16 (3H each, each s, 2 × CH_3_-Ph), 2.26 (2H, t, *J* = 7.3 Hz, H-2), 2.22‒2.12 (2H, m, H-7′/H-9′), 1.99‒1.84 (2H, m, H-6′/H-10′), 1.70‒1.60 (2H, ddd, *J* = 13.5, 6.5, 2.9 Hz, H′-6′/H′-10′), 1.58‒1.35 (6H, m, H-3, H-5 and H′-7′/H′-9′), 1.31‒1.19 (2H, m, H-4); ^13^C NMR (CDCl_3_, 75 MHz) δ 178.0 (C-1), 174.4 (NCO), 173.3 (C-4′), 135.0 (C-5 Ph), 134.9 (C-1 Ph), 132.0 (C-6), 130.5 (C-2), 129.8 (C-3 Ph), 129.3 (C-4 Ph), 106.0 (C-3′), 77.8 (C-8′), 71.4 (C-6), 60.9 (C-5′), 56.0 (OCH_3_), 34.1 (C-2), 32.8 and 32.2 (C-6′ and C-10′), 29.1 (C-5), 28.5 and 28.6 (C-7′ and C-9′), 25.2 (C-4), 24.4 (C-3), 21.0 and 19.7 (2 × CH_3_-Ph); HRMS (TOF–MS ES+) calcd for C_24_H_34_NO_5_ [M + H]^+^ 416.2431, found 416.2442.

### Preparation of active esters of haptens SPm and SPh

Haptens SP*m* and SP*h* were activated through formation of the corresponding *N*-hydroxysuccinimidyl ester using 1-ethyl-3-(3-dimethylaminopropyl)carbodiimide hydrochloride (EDC·HCl) and *N*-hydroxysuccinimide (NHS) in dry acetonitrile (Fig. 2) according to procedure described in the Supplementary Information file. Confirmation of the structure of the active esters was obtained from ^1^H NMR spectroscopic analysis.

*SPm-NHS ester:* 8.3 mg (17.5 µmol) from 8.0 mg of hapten SP*m* (80% yield). ^1^H NMR (CDCl_3_, 300 MHz) δ 7.13 (1H, d, *J* = 7.7 Hz, H-5 Ph), 7.04 (1H, dd, *J* = 7.7, 1.7 Hz, H-6 Ph), 6.96 (1H, d, *J* = 1.7 Hz, H-2 Ph), 6.29 (1H, br s, NH), 3.99 (2H, q, *J* = 7.1 Hz, CH_3_CH_2_OCO_2_), 3.39 (3H, s, C8′-OCH_3_), 3.24 (1H, tt, *J* = 10.6, 4.1 Hz, H-8′), 2.83 (4H, s, OCCH_2_CH_2_CO), 2.59 (1H, t, *J* = 7.5 Hz, H-6), 2.57 (1H, t, *J* = 7.6 Hz, H-2), 2.23 (3H, s, CH_3_-Ph), 2.26‒2.16 (2H, m, H-7′/H-9′), 1.93 (2H, td, *J* = 13.6, 3.7 Hz, H-6′/H-10′), 1.82‒1.71 (2H, m, H′-6′/H′-10′), 1.67‒1.53 (4H, m, H-3 and H-5), 1.50‒1.29 (4H, m, H′-7′/H′-9′ and H-4), 1.08 (3H, t, *J* = 7.1 Hz, CH_3_CH_2_OCO_2_).

*SPh-NHS ester*: 10.9 mg (26.2 µmol) from 12 mg of hapten SP*h* (89% yield). ^1^H NMR (CDCl_3_, 300 MHz) δ 7.08 (1H, d, *J* = 7.7 Hz, H-3 Ph), 7.02 (1H, dd, *J* = 7.7, 1.5 Hz, H-4 Ph), 7.00 (1H, d, *J* = 1.5 Hz, H-6 Ph), 5.93 (1H, br s, NH), 3.72 and 3.62 (1H each, each two dt, *J* = 10.2, 6.1 Hz, H-6 and H′-6), 3.39 (3H, s, OCH_3_), 3.25 (1H, tt, *J* = 10.7, 4.1 Hz, H-8′), 2.83 (4H, m, OCCH_2_CH_2_CO), 2.56 (2H, t, *J* = 7.3 Hz, H-2), 2.29 and 2.17 (3H each, each s, 2 × CH_3_-Ph), 2.25‒2.18 (2H, m, H-7′/H-9′), 1.93 (2H, tdd, *J* = 13.7, 7.6, 3.8 Hz, H-6′/H-10′), 1.71–1.58 (4H, m, H′-6′/H′-10′ and H-3), 1.56–1.46 (2H, m, H-5), 1.41–1.26 (4H, m, H′-7′/H′-9′ and H-4).

### Protein conjugate preparation

Preparation of conjugates of hapten SP*o* was described in a previous article^19^. Concerning SP*m* and SP*h* conjugates, compounds SP*m*-NHS and SP*h*-NHS were dissolved in DMF (50 mM) and coupled to BSA, OVA, and HRP in 100 mM phosphate buffer, pH 7.4, as described in the Supplementary Information file. A small aliquot of each conjugate solution was dialysed using Slide-A-Lyzer MINI dialysis units from Thermo Scientific. Hapten density of protein conjugates was determined by MALDI-TOF‒MS analysis in positive linear mode (1500 shots for each position) in a mass range of 10,000‒120,000 m/z, as described in the Supplementary Information file.

### Antibody generation

Two groups of four 8-week old female Balb/c mice were intraperitoneally immunized; one with conjugate BSA‒SP*m* and the other with conjugate BSA‒SP*o*. Experimental design of the immunization procedures was approved by the Bioethics Committee of the University of Valencia. The European Directive 2010/63/EU and the Spanish laws and guidelines (RD1201/2005 and 32/2007) were followed for animal manipulation. Monoclonals were purified from late stationary-phase culture supernatants by salting out with ammonium sulphate and subsequent affinity chromatography, and they were stored as ammonium sulphate precipitates at 4 °C. Further details can be found in the Supplementary Information file.

### Direct competitive ELISA

Microplates were precoated with GAM in order to immobilise the mAb. The competitive step was carried out by mixing the analyte solution or diluted sample with the enzyme tracer solution. Standard curves were built in borosilicate glass tubes from 0.03 to 30 nM by a threefold serial dilution of the most concentrated standard solution, and a blank (no analyte) was also run. Concentrated stock solutions of the analytes in anhydrous DMF were used to prepare the most concentrated standard solution—the DMF concentration was always lower than 0.1% (v/v). The complete immunoassay procedure can be found in the Supplementary Information file.

### HPLC‒MS/MS analysis

SP, SP-enol, SP-keto, and SP-mono standard solutions were prepared by serial dilution in acetonitrile from 1 to 2500 μg/L in amber glass vials and kept at − 20 °C. The injection volume was 5 µL and the column temperature was 45 °C. The analysis was carried out using a C18 reverse phase column with a gradient of 0.2% (v/v) formic acid in water (A) and acetonitrile (B) as mobile phase at a flow rate of 0.3 mL/min. The elution profile was 0‒3 min, from 5% B to 95% B, 3.0‒3.5 min, 95% B, 3.5‒3.6 min, from 95% B to 5% B, and 3.6‒8 min, 5% B. Spectra were acquired in positive ionization multiple reaction monitoring mode with interchannel delay of 0.02 s.

### Lateral flow immunoassay

The test and control lines on the nitrocellulose membrane were prepared by applying BSA‒SP*h* conjugate and GAM, respectively. The GAM and BSA conjugate solutions—at 1 and 0.5 mg/mL, respectively—were dispensed at 0.5 µL/cm. Then, the membranes were fixed on a 30 cm backing card, and the absorbent and sample pads were fixed. Finally, 4-mm strips were cut with the aid of a guillotine, and they were stored in opaque plastic tubes with desiccating agent at 4 °C. Colloidal GAM-coated GNP were diluted with 10 mM HEPES, pH 7.4, to OD 1.0, and 5 μL of monoclonal SP*m*#23 solution in BioStab was added over 1 mL of GNP dilution up to a final mAb concentration of 0.5 µg/mL. The mixture was incubated at room temperature during 1 h, and Tween-20 was added to reach a concentration of 0.05% (v/v). The obtained gold bioconjugate was stored at 4 °C.

Concentrated stock solutions of the analytes in anhydrous DMF were used to prepare the standard solution at the highest concentration. SP and SP-enol standard curves were built from 0.01 to 10 µg/L by threefold serial dilution in buffer from the most concentrated standard, and a blank (no analyte) was also run. The GNP‒mAb conjugate (10 µL) was mixed with the diluted sample or standard solution in buffer (90 µL) in a microplate well, and the immunochromatographic strip was immediately inserted into the well. The chromatography was allowed to proceed vertically, and after 10 min at room temperature the sample pad was detached from the strip to stop the flow. Then, the strips were dried with a gentle cold air current.

### Food sample preparation

Grapes from local markets were homogenized with a mixer and extracted using the QuEChERS methodology as described by Mohapatra et al^17^. Briefly, 15 mL of acetonitrile was added to 15 g of homogenized sample in a 50-mL polypropylene tube. The mixture was vigorously stirred during 1 min with the aid of a vortex stirrer, and 1.5 g of sodium acetate and 6.0 g of anhydrous magnesium sulphate were added. The mixture was stirred again for 2 min. After centrifugation for 10 min at 2200×*g*, 12 mL of the organic phase was transferred to a 50-mL tube containing 600 mg of primary-secondary amines and 1.8 g of anhydrous magnesium sulphate. The mixture was vortexed during 1 min and centrifuged 10 min at 2200×*g*. Finally, the organic extracts were kept at − 20 °C. Grape extracts were treated with 40 mM NaOH solution during 10 min at room temperature before cELISA analysis. Then, samples were 1:1 diluted in dilution buffer (200 mM Tris·HCl buffer, pH 8.0, containing 280 mM NaCl).

Wine and grape juice samples from local supermarkets were diluted in a 40 mM NaOH solution and incubated 10 min at room temperature. The mixtures were 1:1 diluted in dilution buffer, and a 1-mL aliquot was cleaned-up with 30 mg of PVPP by strong stirring with the aid of a vortex during 2 min. Then, samples were centrifuged at maximum speed during 5 min with a microtube centrifuge, and the supernatant was collected for immunoanalysis.

### Data analysis

The absorbance of each microplate well was immediately read at 492 nm using a reference wavelength at 650 nm, and values were processed with KCJunior software from BioTek Instruments. The SigmaPlot Version 14.0 was used to fit the experimental values to a standard four-parameter logistic curve. Immunoassay sensitivity was estimated as the concentration of analyte affording a 50% decrease (IC_50_) of the maximum absorbance (A_max_) obtained in the absence of analyte. The LOD of the assay was defined as the IC_10_ of the sigmoidal inhibition curve. The LOQ was experimentally determined as the lowest analyte concentration that provided accurate and precise results using fortified food samples.

The RGB (red–green–blue) signal of control and test lines of immunostrips were read with a digital scanner, and data were processed with the ImageJ software from the US National Institutes of Health. The T/C signal value was calculated from the quotient between the test line signal and the control line signal. The cut-off response value was determined according to the following formula for methods with inversely proportional response with the analyte concentration:

$${\text{Cut-off}} = {\text{R}}_{\text{STC}} + t{\text{-value}}_{(0.0{5})}*{\text{SD}}_{\text{STC}}$$

where R_STC_ is the mean T/C value of the samples containing the target analyte at the STC, *t*-value is the one-tailed *t*-test value for a rate of false negative results of 5%, and SD_STC_ is the standard deviation of R_STC_^28^. To calculate the rate of false suspect results, the *t*-value for semi-quantitative methods with inversely proportional response with the analyte concentration was determined as follows:

$$t{\text{-value}} = \left( {\text{mean}}_{\text{blank}} - {\text{cut-off}} \right)/{\text{SD}}_{{\text{blank}}}$$

where mean_blank_ is the mean T/C value obtained for the blank samples and SD_blank_ is the corresponding standard deviation. The obtained *t*-value was used to determine the false suspect rate for a one-tailed distribution using the DIST-T function in Microsoft Excel software.

## Supplementary information

Supplementary Information.

## Data Availability

After signing a material transfer agreement, limited amounts of the monoclonal antibodies and bioconjugates herein described are available for evaluation purposes upon request to the corresponding author.
