# Supplementary material for: Enzyme and lateral flow monoclonal antibody-based immunoassays to simultaneously determine spirotetramat and spirotetramat-enol in foodstuffs
Source: Sci Rep. 2021 Jan 19;11:1809. doi: 10.1038/s41598-021-81432-z (PMC7815808; doi:10.1038/s41598-021-81432-z)

Supplementary Information

Enzyme and lateral flow monoclonal antibody-based immunoassays to simultaneously determine spirotetramat and spirotetramat-enol in foodstuffs

Ramón E. Cevallos-Cedeño, Consuelo Agulló, Antonio Abad-Fuentes, Antonio Abad-Somovilla, and Josep V. Mercader

Contents **Page**

Contents	Page
1. General techniques and equipment	2
2. Hapten synthesis	
2.1. Preparation of synthetic intermediate of hapten SP*m* (Figure S1)	4
2.2. Preparation of hapten SPm	8
2.3. Preparation of hapten SP*h*	11
3. Hapten activation	11
4. Bioconjugate preparation and analysis (Figures S2-S4)	12
5. Antibody generation and selection	14
6. Antibody specificity (Table S1)	15
7. Direct ELISA procedure	15
8. Influence of pH and ionic strength (Figure S5)	15
9. Influence of ethanol and acetonitrile (Figure S6)	16
10. Matrix effects (Figure S7)	16
11. Immunoassay validation by method comparison (Table S2)	17
12. Optimization of the lateral flow immunoassay (Figures S8-S10)	18
13. Validation of immunochromatographic tests (Figure S11 and Table S3)	19
14. ^{1}H NMR spectra of haptens SP*m* and SP*h*	19

1. General techniques and equipment

All solvents were purified by distillation and, if required, they were dried according to standard methods.[1] Reactions were monitored by thin-layer chromatography (TLC) on precoated silica plates (0.25 mm layer thickness, Silica Gel 60 F_{254}) using UV light as the visualizing agent and ethanolic phosphomolybdic acid or aqueous ceric ammonium molybdate solutions and heat as developing agents. Chromatography refers to flash column chromatography and it was carried out with the indicated solvents on silica gel 60 (particle size 0.040–0.063 mm). Melting points were determined using a Kofler hot-stage apparatus and are uncorrected. IR spectra were measured as KBr pellets or liquid films using a Nicolet Avatar 320 spectrometer. IR bands are classified as strong (s), medium (m), or weak (w), depending on their relative intensities in the IR spectrum. Proton and carbon nuclear magnetic resonance (^1H and ^{13}C NMR) spectra were recorded at room temperature (rt) on a Bruker Avance DPX300 spectrometer operating at 300.1 and 75.5 MHz, respectively. The spectra were referenced to residual proton-solvent references (^1H: $CDCl_3$: 7.26 ppm, DMSO-d_6: 2.50 ppm; ^{13}C: $CDCl_3$: 77.16 ppm, DMSO-d_6: 39.52 ppm). Carbon substitution degrees were established by DEPT pulse sequences. The abbreviation used for NMR data are as follows: s =singlet, d = doublet, t = triplet, q = quadruplet, quint = quintuplet, dd: double doublet, dt = double triplet, dq: double quadruplet, tt = *triplet* of *triplets*, ddd = double doublet of doublets, ddt: double doublet of triplets, tdd: *triplet* of double doublets, br = broad, m = multiplet; Ph = phenyl ring. High resolution mass spectra (HRMS) were recorded by the electrospray (ES) ionization mode, which was obtained with a Q-TOF premier mass spectrometer with an electrospray source (Waters, Manchester, UK). The obtained data are expressed as mass/charge ratio (*m/z*).

Biological reagents and media were as those previously described.[2] Briefly, horseradish peroxidase (HRP), ovalbumin (OVA), and Freund's adjuvants were from Merck. Bovine serum albumin (BSA) fraction V and hybridoma fusion and cloning supplement (HFCS) were obtained from Roche Applied Science. P3-X63-Ag 8.653 mouse plasmacytoma cell line was acquired from the European Collection of Cell Cultures. Cell culture media (high-glucose Dulbecco's modified Eagle's medium, DMEM), gentamicin solution, and hypoxanthine–thymidine (HT) and hypoxanthine–aminopterine–thymidine (HAT) supplements were purchased from Gibco BRL. Poly(ethylene glycol) (PEG1500), foetal

[1] D. D. Perrin, W. L. F. Armarego, "Purification of Laboratory Chemicals", 4th ed.; Butterworth Heinemann Press: Oxford (1996).

[2] E. Ceballos-Alcantarilla, C. Agulló, A. Abad-Somovilla, A. Abad-Fuentes, J. V. Mercader. Highly sensitive monoclonal antibody-based immunoassays for the analysis of fluopyram in food samples. *Food Chem.* **288**, 117–126 (2019).

bovine serum (FBS), 200 mM alanyl–glutamine solution, red blood cell lysing buffer Hybri-Max, and MEM non-essential amino acid solution were obtained from Merck. Sephadex G-25 HiTrap Desalting columns and Sepharose HiTrap Protein G HP columns for protein–hapten conjugate purification and for antibody purification, respectively, operated under an ÄKTA Purifier workstation, were obtained from GE Healthcare. HEPES was from PanReac AppliChem. Isotyping reagents for mouse monoclonal antibodies, BioStab antibody stabilizer, *o*-phenylenediamine, and polyvinylpolypyrrolidone were acquired from Merck. Peroxidase labelled rabbit anti-mouse immunoglobulin polyclonal antibody (RAM–HRP) was from Dako. Goat anti-mouse immunoglobulins polyclonal antibody (GAM) was from Jackson ImmunoResearch Laboratories Inc. Primary/secondary amine from Varian and organic solvents from Scharlab were used for sample preparation.

ELISA equipment was as described previously.[2] Briefly, tests were carried out with Costar 96-well flat-bottom high-binding polystyrene ELISA plates from Corning. Microplate wells were washed with an ELx405 washer from BioTek Instruments. Immunoassay absorbance values were read with a PowerWave HT microplate reader also from BioTek. A De'Longhi mixer, a vortex mixer Ms2 from IKA, and a Rotofix 32A centrifuge from Hettich were employed for the extraction of grape samples.

MALDI and LC-MS instruments were those used in previous studies.[2] Briefly, a 5800 matrix-assisted laser desorption ionization time-of-flight (MALDI-TOF/TOF) MS apparatus from AB Sciex was used for bioconjugate analysis. SP residues and its metabolites were determined by HPLC using a UPLC Acquity TQD system from Waters (Milford, MA) furnished with a binary solvent delivery system, an autosampler, and a BEH C18 (1.7 µm, 2.1 × 50 mm) column also from Waters. An Acquity triple quadrupole MS detector, also from Waters, with a Z-spray electrospray ionization source (3 kV capillary voltage, and 120 °C and 300 °C as source and desolvation temperature, respectively) were employed for tandem mass acquisitions.

Backed high-binding nitrocellulose membranes (25 mm wide and 15 µm pore size) from MDI Advanced Microdevices PVT Ltd. (Ambala Cantt, India), cellulose sample pad (17 mm wide) from Millipore Corporation (Billerica, MA), cellulose absorbent pad (43 mm wide) from Ahlstrom-Munksjö (Helsinki, Finland), and 8×30 cm backing cards from Kenosha (Amstelveen, The Netherlands) were used to build the strips for lateral flow immunochromatography. GAM-coated 40 nm gold nanoparticles (GNP), OD 10, were procured from BBI Solutions (Crumlin, UK). A BioDot ZX1010 platform equipped with two Frontline HR microliter contact dispensers (Irvine, CA) was employed to dispense the immunoreagents on the membrane. Strips were cut with a CM5000 guillotine cutter, also from BioDot.

Signal from lateral flow assays was read using an EPSON Perfection V39 ultra-compact colour image scanner of Seiko Epson Corp. (Suwa, Japan).

2. Hapten synthesis

*2.1. Synthesis of aryl iodide **1***

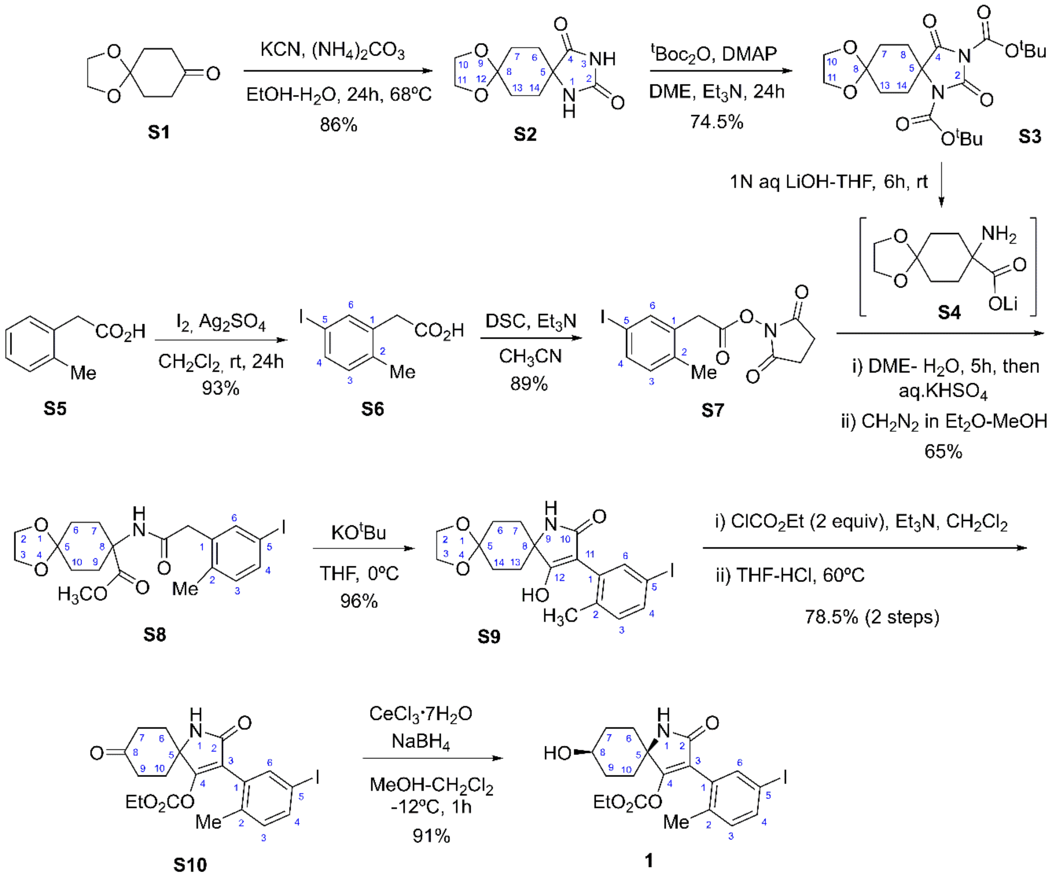

Figure S1. Preparation of aryl iodide **1**, a synthetic intermediate of hapten SP*m*.

Preparation of 9,12-dioxa-1,3-diazadispiro[4.2.4⁸.2⁵]tetradecane-2,4-dione (**S2**). A solution of 1,4-cyclohexanedione monoethylene acetal (**S1**, 4.0 g, 25.6 mmol), KCN (1.67 g, 25.6 mmol, 1 equiv) and $(NH_4)_2CO_3$ (6.24 g, 65 mmol, 2.5 equiv) in a 1:1 mixture of EtOH-H_2O (87 mL) was stirred at 68 °C for 24 h. The reaction mixture was cooled to rt and then allowed to stand in the fridge overnight. The solid was filtered off, washed with cold water and dried to hydantoin **S2** (3.59 g). A second crop (1.39 g) was obtained from the filtrate after standing in the fridge for 24 h (the overall yield of **S2** obtained was 86%). Mp 240.5-242.3 °C (crystallized from H_2O), Lit.[3] 240-242 °C (crystallized from EtOH). IR ν_{max} (cm^{-1}) 3189 (s), 3069 (m), 2966 (s), 2762 (m), 1771 (s), 1732 (s), 1414 (m), 1101 (m), 892 (m), 756 (m); ^{1}H NMR

[3] M. Oba, N. Ishikawa, Y. Demizu, M. Kurihara, H. Suemune, M. Tanaka. Helical oligomers with a changeable chiral acetal moiety. *Eur. J. Org. Chem.*, 7679–7682 (2013).

(DMSO-d_6, 300 MHz) δ 10.62 (1H, br s, NH), 8.44 (1H, s, NH), 3.86 (4H, s, OCH_2CH_2O), 1.90-1.50 (8H, m); ^{13}C NMR (DMSO-d_6, 75 MHz) δ 178.3 (C-4), 156.4 (C-2), 106.8 (C-8), 63.7 and 63.6 (OCH_2CH_2O), 61.0 (C-5), 31.3 (C-7/C-9), 29.8 (C-6/C-10).

Preparation of di-tert-butyl 2,4-dioxo-9,12-dioxa-1,3-diazadispiro[4.2.4^8.2^5]tetradecane-1,3-dicarboxylate (**S3**). Anhydrous Et_3N (635 µL, 4.56 mmol) was added to a mixture of hydantoin **S2** (1.032 g, 4.56 mmol), di-*tert*-butyl dicarbonate (3.485 g, 15.97 mmol) and (dimethylamino)pyridine (21 mg, 0.17 mmol) in anhydrous dimethoxyethane (23 mL) at rt under N_2. The mixture was stirred at rt overnight and filtered to separate the white solid formed. The yellowish filtrate and washing were transferred to a round-bottom flask and concentrated at reduced pressure to eliminate solvents. The obtained residue was dissolved in $CHCl_3$ and washed with H_2O and brine, dried over anhydrous Na_2SO_4 and concentrated under vacuum to afford compound **S3** (1.450 g, 74.5%) as a solid, whose ^1H NMR spectrum showed that it had adequate purity (>95%) to be used in the next stage without further purification. Mp 195-198 °C (crystallized from benzene); IR ν_{max} (cm^{-1}) 2987 (m), 2822 (m), 1775 (s), 1716 (s), 1370 (s), 1262 (s), 1143 (s), 1036 (m), 885 (m); ^1H NMR ($CDCl_3$, 300 MHz) δ 3.96 (4H, s, OCH_2CH_2O), 2.91 (2H, td, *J* = 13.8, 4.6 Hz, H-6 and H-14), 2.36 (2H, td, *J* = 13.8, 4.6 Hz, H'-6 and H'-14), 1.85-1.66 (4H, m, H-7 and H-13), 1.58 and 1.57 (9H each, each s, $CO_2C(CH_3)_3$ x 2);[2] ^{13}C NMR ($CDCl_3$, 75 MHz), δ 169.5 (C-4), 148.1 (C-2), 147.6 and 145.3 (*CO_2*$C(CH_3)_3$ x 2), 107.2 (C-8), 86.6 and 84.9 (CO_2*C*$(CH_3)_3$), 64.5 and 64.3 (OCH_2CH_2O), 63.2 (C-5), 30.1 (C-7/C-9), 28.1 (C-6/C-10), 27.0 and 27.7 ($CO_2C(CH_3)_3$ x 2).

Preparation of 2-(5-iodo-2-methylphenyl)acetic acid (**S6**). A mixture of 2-(*o*-tolyl)acetic acid (**S5**, 2.0 g, 13.32 mmol), iodine (3.381 g, 13,32 mmol) and Ag_2SO_4 (4.15 g, 13.32 mmol) was suspended in anhydrous CH_2Cl_2 (34.5 mL) and the heterogeneous mixture was stirred at rt for 24 h. La mixture was filtered, washing with CH_2Cl_2, and the combined filtrate and washing were washed with a 10% aqueous solution of $NaHSO_3$ and brine, dried over anhydrous $MgSO_4$ and concentrated at reduced pressure to give an approximately 95:15 mixture of aryl iodide **S6** and regioisomeric 2-(3-iodo-2-methylphenyl)acetic acid (3.432 g, 93%) as a solid. It was used as such in the next step. IR ν_{max} (cm^{-1}) 2500-3550 (broad, s), 3012 (w), 2915 (w), 1707 (s), 1586 (w), 1555 (w), 1403 (m), 1222 (m); ^1H NMR ($CDCl_3$, 300 MHz), only the signals of the major regioisomer are given, δ 7.53 (1H, br s, H-6), 7.51 (1H, dd, *J* = 7.8, 1.8 Hz, H-4), 6.93 (1H, d, *J* = 7.8 Hz, H-3), 3.61 (2H, s, CH_2), 2.26 (3H, s, CH_3);[4] ^{13}C NMR ($CDCl_3$,

[4] R. Fischer, T. Bretschneider, K. Ilg, S. Lehr, D. Feucht, O. Malsam, U. Reckmann, G. Bojack, C. Arnold, T. Auler, M. Hills, J. Martin, H. Kehne, W. Hempel, E. Sanwald. Iodophenyl-substituted cyclic keto enols and their preparation, agrochemical compositions, and use as pesticides and/or herbicides. Patent nº WO 2006029799 A1, March 23 (2006).

75 MHz), only the signals of the major regioisomer are given, δ 177.5 (CO_2H), 138.8 (C-6), 136.6 (C-4), 134.2 (C-2), 132.8 (C-1), 132.2 (C-3), 90.7 (C-5), 38.5 (CH_2), 19.2 (CH_3).

Preparation of methyl 8-(2-(5-iodo-2-methylphenyl)acetamido)-1,4-dioxaspiro[4.5]decane-8-carboxylate (**S8**).

i) Acid **S6** (1.008 g, 3.6 mmol) and *N*,*N*'-disuccinimidyl carbonate (1.2 g, 4.68 mmol) were dissolved in MeCN (35.5 mL) under N_2 at rt. Dry Et_3N (1.7 mL, 12.84 mmol) was then added and the resulting solution was stirred overnight at rt. The reaction mixture was diluted with water and extracted with EtOAc, the combined organic layers were washed with a 10% aqueous solution of $NaHCO_3$ and brine, dried over anhydrous Na_2SO_4 and concentrated at reduced pressure. The residue obtained (1.328 g) was purified by chromatography, using $CHCl_3$ as eluent, to afford the *N*-hydroxysuccinimidyl ester **S7** (1.212 g, 89%) as a viscous oil that solidified on standing. Mp 121-123 °C (crystallized from Et_2O); IR ν_{max} (cm^{-1}) 3038 (w), 2951 (w), 1807 (s), 1778 (s), 1740 (s), 1585 (w), 1555 (w), 1204 (s), 1065 (s), 645 (m); ^1H NMR ($CDCl_3$, 300 MHz) δ 7.60 (1H, br s, H-6), 7.53 (1H, br d, *J* = 8.1 Hz, H-4), 3.86 (2H, s, CH_2), 2.82 (4H, $COCH_2CH_2CO$), 2.29 (3H, s, CH_3).

ii) A solution of $LiOH{\cdot}H_2O$ (319 mg, 7.60 mmol) in water (7.6 mL) was dropwise added to a solution of hydantoin **S3** (810 mg, 1.90 mmol) in THF (29 mL) at rt during 6 h. The reaction mixture was filtered to remove insoluble material and the filtrate was concentred at reduced pressure to give the lithium salt of 8-amino-1,4-dioxaspiro[4.5]decane-8-carboxylic acid (**S4**) as a solid.

A solution of the *N*-hydroxysuccinimidyl ester **S7** prepared above (1.063 g, 2.85 mmol, 1.5 equiv) in dimethoxyethane (7.6 mL) was dropwise added to a stirred solution of the lithium salt **S4** (about 1.90 mmol based on the starting hydantoin) in a mixture of dimethoxyethane (7.6 mL) and H_2O (7.6 mL) and the resulting yellowish mixture was stirred at rt until no *N*-hydroxysuccinimidyl ester **S7** was observed by TLC (eluent $CHCl_3$-MeOH 9:1; about 3-4 h). The reaction mixture was cooled in an ice-water bath, acidified to pH 2-3 with 1 M aqueous $KHSO_4$ and extracted with Et_2O. The combined organic layers were washed with water and brine and dried over Na_2SO_4. The residue obtained after evaporation of the solvent was dissolved in a 2:1 mixture of Et_2O-MeOH and treated with excess CH_2N_2 (1 h, rt). The solvents were evaporated at reduced pressure and the obtained residue was chromatographed, using $CHCl_3$ as eluent, to afford amide **S8** (584 mg, 65%) as a semisolid. IR ν_{max} (cm^{-1}) 3325 (s), 2950 (s), 2874 (s), 1779 (s), 1739 (s), 1651 (s), 1527 (s), 1103 (s), 908 (m), 731 (s); ^1H NMR ($CDCl_3$, 300 MHz) δ 7.48 (1H, br s, H-6 Ph), 7.47 (1H, dd, *J* = 8.4, 1.8 Hz, H-4 Ph), 6.89 (1H, d, *J* = 8.4 Hz, H-3 Ph), 5.38 (1H, NH), 3.86 (4H, s, $COCH_2CH_2CO$), 3.65 (3H, s, CO_2CH_3), 3.44 (2H, s, $NCOCH_2$), 2.19 (3H, CH_3-Ph), 2.09 (2H, ddd, *J* = 14.5, 11.5, 4.3 Hz, H-7/H-9), 1.93 (2H, dt, *J* = 14.5, 4.3 Hz, H'-7/H'-9), 1.63

(2H, dt, J = 12.7, 4.3 Hz, H-6/H-10), 1.42 (2H, m, H'-6/H'-10); ^{13}C NMR ($CDCl_3$, 75 MHz) δ 174.0 ($\mathit{C}O_2CH_3$), 170.5 (CONH), 139.1 (C-6 Ph), 137.3 (C-2 Ph), 137.1 (C-4 Ph), 136.2 (C-1 Ph), 132.9 (C-3 Ph), 107.8 (C-5), 91.5 (C-5 Ph), 64.8 and 64.7 (OCH_2CH_2O), 58.43 (C-8), 52.9 ($CO_2\mathit{CH_3}$), 41.5 ($NCO\mathit{CH_2}$), 30.8 and 30.6 (C-7/C-9 and C-6/C-10), 19.5 (CH_3); HRMS (TOF MS ES+) calcd for $C_{19}H_{25}INO_5$ $[M+H]^{\cdot+}$ 474.0772, found 474.0775.

Preparation of 12-hydroxy-11-(5-iodo-2-methylphenyl)-1,4-dioxa-9-azadispiro[4.2.4^8.2^5] tetradec-11-en-10-one (**S9**). A solution of amide **S8** (295 mg, 0.623 mmol) and potassium *tert*-butoxide (147.7 mg, 1.557 mmol) in anhydrous THF (15 mL) was stirred for 40 min at rt under N_2, then diluted with water, cooled in and water-ice bath, acidified with a 10% aqueous solution of $KHSO_4$ and extracted with Et_2O. The combined organic layers were washed with brine, dried over anhydrous $MgSO_4$ and concentrated at reduced pressure to give compound **S9** (263.5 mg, 96%) as a slightly coloured solid. Mp 179 °C with decomposition (crystallized from benzene); IR ν_{max} (cm^{-1}) 3372 (m), 2932 (m), 2883 (m), 1655 (s), 1612 (s), 1576 (m), 1313 (m), 1087 (s), 793 (m); ^{1}H NMR (DMSO-d_6, 300 MHz) δ 10.95 (1H, s, OH), 8.23 (1H, s, NH), 7.52 (1H, dd, J = 8.1, 1.9 Hz, H-4 Ph), 7.39 (1H, d, J = 1.9, H-6 Ph), 7.02 (1H, d, J = 8.1 Hz, H-3 Ph), 3.88 (4H, s, OCH_2CH_2O), 2.08 (3H, s, CH_3), 2.17-2.04 (2H, m, H-7/H-14), 1.87 (2H, td, J = 13.7, 3.6 Hz, H'-7/H'-14), 1.69 (2H, br d, J = 12.8 Hz, H-6/H-14), 1.40 (2H, br d, J = 12.8 Hz, H'-6/H'-14); ^{13}C NMR (DMSO-d_6, 75 MHz) δ 171.2 (CONH), 139.0 (C-4 Ph), 137.4 (C-2 Ph), 135.5 (C-1 Ph), 131.7 (C-3 Ph), 128.3 (C-6 Ph), 107.2 (C-11), 90.1 (C-5 Ph), 63.63 and 63.58 (OCH_2CH_2O), 59.1 (C-8), 31.6 and 30.7 (C-6/C-14 and C-7/C-13), 19.2 (CH_3); HRMS (TOF MS ES+) calcd for $C_{18}H_{21}INO_4$ $[M+H]^{\cdot+}$ 442.0510, found 442.0505.

Preparation of ethyl (3-(5-iodo-2-methylphenyl)-2,8-dioxo-1-azaspiro[4.5]dec-3-en-4-yl) carbonate (**S10**). Et_3N (139 mg, 191 µL, 1.371 mmol) and $ClCO_2Et$ (124 mg, 108 µL, 1.192 mmol) were successively added to a solution of enol **S9** (263 mg, 0.596 mmol) in anhydrous CH_2Cl_2 (20 mL) at 0 °C under N_2. The mixture was allowed to warm to rt and stirred for 2 h, then diluted with CH_2Cl_2 and successively washed with 0.5 M HCl, 5% aqueous $NaHCO_3$ and brine, dried over anhydrous Na_2SO_4, and concentrated. The residue obtained (290 mg) was dissolved in THF (6 mL), aqueous 1 M HCl (4 mL) was added and the mixture stirred at 60 °C during 2 h. The reaction mixture was cooled to rt and extracted with EtOAc, the combined organic extracts were washed with 5% aqueous $NaHCO_3$ and brine and dried over anhydrous $MgSO_4$. The oily residue obtained after evaporation of the solvent under vacuum was purified by chromatography, using $CHCl_3$ and 94:6 $CHCl_3$-EtOAc as eluents, to give ketone **S10** (220 mg, 78.5%). IR ν_{max} (cm^{-1}) 3230 (m), 3071 (w), 2932 (w), 1779 (m), 1695 (s), 1204 (s), 755 (w); ^{1}H NMR ($CDCl_3$, 300 MHz) δ 9.55 (1H, s, NH), 7.50 (1H, dd, J = 8.1, 1.9 Hz, H-4 Ph), 7.40 (1H, d, J = 1.9 Hz, H-6 Ph), 6.92 (1H, d, J = 8.1 Hz, H-3 Ph), 4.00 (2H, q, J = 7.1 Hz, $CH_3\mathit{CH_2}OCO_2$), 2.68 (2H, td, J = 14.5, 6.0 Hz,

H-7/H-9), 2.44 (2H, br d, *J* = 14.5 Hz, H'-7/H'-9), 2.34-2.19 (2H, m, H-6/H-10), 2.15 (3H, s, CH_3), 1.98 (2H, m, H'-6/H'-10), 1.09 (3H, t, *J* = 7.1 Hz, $CH_3CH_2OCO_2$); ^{13}C NMR ($CDCl_3$, 75 MHz) δ 208.5 (C-8), 171.0 (CONH), 165.0 (C-4), 149.9 (OCO_2), 137.9 (C-4 Ph), 137.8 (C-6 Ph), 137.4 (C-1 Ph), 132.5 (C-3 Ph), 130.5 (C-2 Ph), 120.8 (C-3), 90.1 (C-5 Ph), 66.3 ($CH_3CH_2OCO_2$), 60.8 (C-5), 37.9 (C-7/C-9), 33.8 (C-6/C-10), 19.6 (CH_3-Ph), 13.9 ($CH_3CH_2OCO_2$); HRMS (TOF MS ES+) calcd for $C_{19}H_{21}INO_5$ $[M+H]^{\cdot+}$ 470.0459, found 470.0452.

Preparation of ethyl (8-hydroxy-3-(5-iodo-2-methylphenyl)-2-oxo-1-azaspiro[4.5]dec-3-en-4-yl) carbonate (**1**). The followed experimental procedure was similar to that previously used for the preparation of related compounds.[5] $CeCl_3\cdot7H_2O$ (382 mg, 1.028 mmol) was added to a solution of ketone **S10** (193 mg, 0.411 mmol) in 3:1 mixture of MeOH and CH_2Cl_2 (14 mL). The resulting solution was cooled to –12 °C and $NaBH_4$ (38.8 mg, 1.027 mmol) was added, and the mixture stirred at the same temperature for 1 h. The mixture was treated with a few drops of acetone and stirred for 5 min, then poured into a saturated aqueous solution of sodium citrate and extracted with EtOAc. The combined organic layers were washed with brine, dried over anhydrous $MgSO_4$ and concentrated at reduced pressure to give alcohol **1** (176.3 mg, 91%) as a white solid. Mp 229-229.5 °C (crystallized from MeOH); IR ν_{max} (cm^{-1}) 3324 (s), 2943 (m), 1770 (s), 1683 (s), 1231 (s), 1058 (m), 651 (m); ^1H NMR (DMSO-d_6, 300 MHz) δ 9.01 (1H, s, NH), 7.60 (1H, dd, *J* = 8.1, 1.9 Hz, H-4 Ph), 7.37 (1H, d, *J* = 1.9 Hz, H-6 Ph), 7.06 (1H, d, *J* = 8.1 Hz, H-3 Ph), 4.71 (1H, d, *J* = 3.2 Hz, OH), 4.02 (2H, q, *J* = 7.1 Hz, $CH_3CH_2OCO_2$), 3.46 (1H, m, H-8), 2.11 (3H, s, CH_3-Ph), 1.80 (4H, m, H-6/H-10), 1.54 (4H, m, H-7/H-9), 1.03 (3H, t, *J* = 7.1, $CH_3CH_2OCO_2$); ^{13}C NMR (DMSO-d_6, 75 MHz) δ 167.9 (CONH), 165.0 (C-4), 149.6 (OCO_2), 137.2 (C-4 Ph), 136.8 (C-6 Ph), 136.7 (C-1 Ph), 132.1 (C-3 Ph), 131.5 (C-2 Ph), 119.4 (C-3), 90.3 (C-5 Ph), 67.4 (C-8), 65.6 ($CH_3CH_2OCO_2$), 60.1 (C-5), 31.7 (C-6/C-10), 30.9 (C-7/C-9), 19.8 (CH_3-Ph), 13.5 ($CH_3CH_2OCO_2$); HRMS (TOF MS ES+) calcd for $C_{19}H_{23}INO_5$ $[M+H]^{\cdot+}$ 472.0615, found 472.0614.

2.2. Synthesis of hapten SPm (Figure 1b)

Preparation of ethyl (3-(5-iodo-2-methylphenyl)-2,8-dimethoxy-1-azaspiro[4.5]deca-1,3-dien-4-yl) carbonate (**2**). Anhydrous $CaSO_4$ (155.2 mg, 1.140 mmol) and Ag_2O (198.1 mg, 0.855 mmol) were sequentially added to a solution of alcohol-amide **1** (120 mg, 0.285 mmol), previously dried under P_2O_5 and vacuum overnight, in anhydrous ICH_3 (1.7 mL) at room temperature, in the dark, under N_2. The heterogeneous reaction mixture was stirred under these conditions for about 48 h, then diluted with EtOAc and filtered through a plug of Celite using EtOAc for washing the solid. The filtrate and washing

[5] Cevallos-Cedeño, R. E., Agulló, C., Abad-Somovilla, A., Abad-Fuentes, A. & Mercader, J. V. Hapten design and antibody generation for immunoanalysis of spirotetramat and spirotetramat-enol. *ACS Omega* **3**(9), 11950–11957 (2018).

were combined and washed with brine, dried over anhydrous $MgSO_4$, and concentrated at reduced pressure. The obtained residue was purified by chromatography, using $CHCl_3$ and 95:5 $CHCl_3$–EtOAc as eluents, to give dimethylated derivative **2** (99.6 mg, 70%) as a viscous oil. IR ν_{max} (cm^{-1}) 2941 (m), 2847 (s), 1774 (s), 1676 (m), 1579 (m), 1215 (s), 1098 (s), 1038 (m), 757 (w); ^1H NMR ($CDCl_3$, 300 MHz) δ 7.53 (1H, dd, *J* = 8.1, 1.9 Hz, H-4 Ph), 7.44 (1H, d, *J* = 1.9 Hz, H-6 Ph), 7.06 (1H, d, *J* = 8.1 Hz, H-3 Ph), 4.07 (2H, q, *J* = 7.1 Hz, $CH_3\mathit{CH_2}OCO_2$), 3.87 (3H, s, C2-OCH_3), 3.40 (3H, s, C8-OCH_3), 3.29 (1H, td, *J* = 10.1, 5.0 Hz, H-8), 2.16 (3H, s, CH_3-Ph), 2.10–2.01 (2H, m, H-6/H-10), 1.99–1.76 (4H, m, H'-6/H'-10 and H-7/H-9), 1.53–1.41 (2H, m, H'-7/H'-9), 1.17 (3H, t, *J* = 7.1 Hz, $\mathit{CH_3}CH_2OCO_2$); ^{13}C NMR ($CDCl_3$, 75 MHz) δ 170.2 (C-2), 167.3 (C-4), 150.4 (OCO_2), 137.8 (C-4 Ph), 137.3 (C-6 Ph), 137.2 (C-1 Ph), 132.1 (C-3 Ph), 131.7 (C-2 Ph), 118.1 (C-3), 90.1 (C-5 Ph), 78.7 (C-8), 70.6 (C-5), 65.6 ($CH_3\mathit{CH_2}OCO_2$), 55.8 and 54.62 (O$\mathit{CH_3}$ × 2), 31.9 (C-6/C-10), 28.5 (C-7/C-9), 19.4 (CH_3-Ph), 14.0 ($\mathit{CH_3}CH_2OCO_2$); HRMS (TOF-MS ES+) calcd for $C_{21}H_{27}INO_5$ $[M+H]^{\cdot+}$ 500.0928, found 500.0924.

Preparation of tert-butyl 6-(3-(4-((ethoxycarbonyl)oxy)-2,8-dimethoxy-1-azaspiro[4.5]deca-1,3-dien-3-yl)-4-methylphenyl)hex-5-ynoate (**4**). Degassed Et_3N (30 µL, 0.215 mmol) was dropwise added under a N_2 atmosphere to a mixture of aryl iodide **2** (88.5 mg, 0.177 mmol), *tert*-butyl hex-5-ynoate (**3**) (45 mg, 0.268 mmol), $Cl_2Pd(PPh_3)_2$ (6.5 mg, 9 µmol), and CuI (3.5 mg, 18 µmol) in anhydrous MeCN (3.3 mL). The mixture was stirred at room temperature for 2.5 h, then diluted with EtOAc, washed with water and brine, dried over anhydrous $MgSO_4$, and concentrated. Purification by chromatography, using 9:1 hexane–EtOAc as eluent, afforded aryl-alkyne **4** (74.5 mg, 78%) as a slightly coloured, viscous oil. ^1H NMR ($CDCl_3$, 300 MHz) δ 7.24 (1H, dd, *J* = 8.0, 1.7 Hz, H-6 Ph), 7.16 (1H, d, *J* = 1.7 Hz, H-2 Ph), 7.12 (1H, d, *J* = 8.0 Hz, H-5 Ph), 4.04 (2H, q, *J* = 7.1 Hz, $CH_3\mathit{CH_2}OCO_2$), 3.86 (3H, s, C2'-OCH_3), 3.40 (3H, s, C8'-OCH_3), 3.29 (1H, td, *J* = 10.1, 4.3 Hz, H-8'), 2.43 (2H, t, *J* = 6.9 Hz, H-4), 2.38 (2H, t, *J* = 7.6 Hz, H-2), 2.20 (3H, s, CH_3-Ph), 2.10–2.00 (2H, m, H-6'/H-10'), 1.99–1.75 (6H, m, H'-6'/H'-10', H-7'/H-9' and H-3), 1.52–1.39 (2H, m, H'-7'/H'-9'), 1.45 (9H, s, CMe_3), 1.14 (3H, t, *J* = 7.1 Hz, $\mathit{CH_3}CH_2OCO_2$); ^{13}C NMR ($CDCl_3$, 75 MHz) δ 172.6 (C-1), 169.7 (C-2'), 167.6 (C-4'), 150.3 (OCO_2), 137.0 (C-4 Ph), 132.4 (C-6 Ph), 130.4 (C-2 Ph), 130.0 (C-5 Ph), 129.2 (C-3 Ph), 121.1 (C-1 Ph), 118.6 (C-3'), 88.6 (C-5), 80.9 (C-6), 80.3 ($\mathit{C}Me_3$), 78.6 (C-8'), 70.3 (C-5'), 65.3 ($CH_3\mathit{CH_2}OCO_2$), 55.6 and 54.3 (O$\mathit{CH_3}$ × 2), 34.4 (C-2), 28.3 (C-7/C-9), 28.1 ($\mathit{CMe_3}$), 24.1 (C-3), 19.5 (CH_3-Ph), 18.8 (C-4), 13.8 ($\mathit{CH_3}CH_2OCO_2$); HRMS (TOF-MS ES+) calcd for $C_{31}H_{42}NO_7$ $[M+H]^{\cdot+}$ 540.2956, found 540.2958.

Preparation of tert-butyl 6-(3-(4-((ethoxycarbonyl)oxy)-2,8-dimethoxy-1-azaspiro[4.5]deca-1,3-dien-3-yl)-4-methylphenyl)hexanoate (**5**). A solution of aryl-alkyne **4** (57.6 mg, 0.106 mmol) and Wilkinson's rhodium catalyst (6.1 mg, 6.5 µmol, 6%) in anhydrous THF (2.5 mL) was evacuated and purged under an atmosphere of hydrogen. The followed experimental procedure was similar to that

previously used for the preparation of related compounds.[5] The hydrogen pressure was regulated to 3.7 bar and the reaction mixture was stirred at room temperature during 24 h. The solvent was removed under reduced pressure and the residue obtained was purified by chromatography, using hexane–EtOAc mixtures from 8:2 to 7:3 as eluent, to give compound **5** (50 mg, 85%) as a colourless oil. IR ν_{max} (cm^{-1}) 2977 (m), 2935 (s) 1775 (s), 1730 (s), 1676 (m), 1580 (s), 1217 (s), 1155 (s), 1101 (s), 1041 (m); ^{1}H NMR ($CDCl_3$, 300 MHz) δ 7.10 (1H, d, *J* = 7.8 Hz, H-5 Ph), 7.02 (1H, dd, *J* = 7.8, 1.8 Hz, H-6 Ph), 6.93 (1H, d, *J* = 1.8 Hz, H-2 Ph), 4.01 (2H, q, *J* = 7.1 Hz, CH_3*CH_2*OCO_2), 3.86 (3H, s, C2'-OCH_3), 3.40 (3H, s, C8'-OCH_3), 3.37–3.21 (1H, m, H-8'), 2.54 (2H, t, *J* = 7.8 Hz, H-6), 2.20 (2H, t, *J* = 7.4 Hz, H-2), 2.17 (3H, s, CH_3- Ph), 2.10-2.01 (2H, m, H-6'/H-10'), 2.00-1.76 (4H, m, H'-6'/H'-10' and H-7'/H-9'), 1.65–1.53 (4H, m, H-3 and H-5), 1.53–1.41 (2H, m, H'-7'/H'-9'), 1.43 (9H, s, CMe_3), 1.39–1.29 (2H, m, H-4), 1.11 (3H, t, *J* = 7.1 Hz, *CH_3*CH_2OCO_2); ^{13}C NMR ($CDCl_3$, 75 MHz) δ 173.3 (C-1), 169.4 (C-2'), 168.0 (C-4'), 150.6 (OCO_2), 139.8 (C-1 Ph), 134.4 (C-4 Ph), 130.1 (C-5 Ph), 129.4 (C-2 Ph), 128.9 (C-3 Ph), 128.5 (C-6 Ph), 119.7 (C-3'), 80.1 (*C*Me_3), 78.8 (C-8'), 70.3 (C-5'), 65.3 (CH_3*CH_2*OCO_2), 55.8 and 54.5 (O*CH_3* × 2), 35.6 (C-6), 35.3 (C-2), 32.0 (C-6'/C-10'), 31.3 (C-3), 28.8 (C-7'/C-9'), 28.5 (C-4), 28.2 (*CMe_3*), 25.1 (C-5), 19.2 (CH_3-Ph), 13.9 (*CH_3*CH_2OCO_2); HRMS (TOF-MS ES+) calcd for $C_{31}H_{46}NO_7$ $[M+H]^{\cdot+}$ 544.3269, found 544.3267.

Preparation of tert-butyl 6-(3-(4-((ethoxycarbonyl)oxy)-8-methoxy-2-oxo-1-azaspiro[4.5]dec-3-en-3-yl)-4-methylphenyl)hexanoate (**6**). Dry Et_3N (21 µL, 147 µmol), $(^tBu)_2Si(OTf)_2$ (36 mg, 26.7 µL, 0.082 mmol), and MeI (5 µL, 0.081 mmol) were successively added to a solution of **5** (40 mg, 73.6 µmol) in anhydrous DMF (0.8 mL) at 0 °C under N_2. The reaction mixture was allowed to warm to room temperature and stirred for 60 h. The resulting yellowish mixture was diluted with water and extracted with Et_2O, the combined organic layers were washed with a 5% (w/v) aqueous solution of LiCl and brine, and dried over anhydrous $MgSO_4$. Chromatographic purification of the residue obtained after evaporation of the solvent at reduced pressure, using $CHCl_3$ and 95:5 $CHCl_3$–MeOH as eluents, gave compound **6** (32 mg, 82%). ^{1}H NMR ($CDCl_3$, 300 MHz) δ 7.11 (1H, d, *J* = 7.8 Hz, H-5 Ph), 7.02 (1H, dd, *J* = 7.8, 1.8 Hz, H-6 Ph), 6.94 (1H d, *J* = 1.8 Hz, H-2 Ph), 6.82 (1H, s, NH), 3.98 (2H, q, *J* = 7.1 Hz, CH_3*CH_2*OCO_2), 3.37 (3H, s, C8'-OCH_3), 3.23 (1H, tt, *J* = 10.7, 4.1 Hz, H-8'), 2.53 (1H, t, *J* = 7.6 Hz, H-6), 2.24–2.13 (2H, m, H-7'/H-9'), 2.22 (3H, s, CH_3-Ph), 2.19 (2H, t, *J* = 7.4 Hz, H-2), 1.91 (2H, td, *J* = 13.6, 3.8 Hz, H-6'/H-10'), 1.81–1.70 (2H, m, H'-6'/H'-10'), 1.65–1.51 (4H, m, H-3 and H-5), 1.50–1.40 (2H, m, H'-7'/H'-9'), 1.43 (9H, s, CMe_3), 1.38–1.28 (2H, m, H-4), 1.07 (3H, t, *J* = 7.1 Hz, *CH_3*CH_2OCO_2); ^{13}C NMR ($CDCl_3$, 75 MHz) δ 173.3 (C-1), 170.1 (NCO), 165.0 (C-4'), 150.0 (OCO_2), 139.8 (C-1 Ph), 134.6 (C-3 Ph), 130.3 (C-5 Ph), 129.4 (C-2 Ph), 128.8 (C-6 Ph), 128.0 (C-4 Ph), 121.9 (C-3'), 80.0 (*C*Me_3), 77.3 (C-8'), 65.8 (CH_3*CH_2*OCO_2), 60.4 (C-5'), 55.9 (O*CH_3*), 35.6 (C-2), 35.3 (C-6), 31.8 (C-6'/C-10'), 31.3 (C-5), 28.9 (C-4),

28.5 (C-7'/C-9'), 28.2 (*CMe*$_3$), 25.1 (C-3), 19.4 (CH_3-Ph), 13.8 (*CH_3*CH_2OCO_2); HRMS (TOF MS ES+) calcd for $C_{30}H_{44}NO_7$ $[M+H]^{\cdot+}$ 530.3112, found 530.3107.

Preparation of 6-(3-(4-((ethoxycarbonyl)oxy)-8-methoxy-2-oxo-1-azaspiro[4.5]dec-3-en-3-yl)-4-methylphenyl)hexanoic acid (**hapten SP*m***). A solution of *tert*-butyl ester **6** (30 mg, 0,057 mmol) in formic acid (0.9 mL) was stirred at room temperature for 4 h. Dry benzene was added and the mixture was concentrated at reduced pressure. This procedure was repeated twice to remove all traces of formic acid to give practically pure hapten SP*m* (26.5 mg, quantitative) as a semisolid.

2.3. Synthesis of hapten SPh (Figure 1c)

Preparation of tert-butyl 6-((-3-(2,5-dimethylphenyl)-8-methoxy-2-oxo-1-azaspiro[4.5]dec-3-en-4-yl)oxy)hexanoate (**8**). A suspension of SP-enol (25 mg, 0.083 mmol), alkyl tosylate **7** (54 mg, 0.158 mmol), and $CsCO_3$ (54 mg, 0.166 mmol) in anhydrous DMF (1 mL) was stirred at room temperature for 36 h under N_2. The reaction mixture was diluted with water and extracted with EtOAc. The organic extracts were combined, washed with a 5% (w/v) aqueous solution of LiCl and brine, dried over anhydrous $MgSO_4$, and concentrated. Purification by chromatography, using $CHCl_3$ and 99:1 $CHCl_3$–MeOH as eluents, afforded enol ether **8** (29.8 mg, 76%) as an oil. IR ν_{max} (cm^{-1}) 3196 (w), 3065 (w), 2937 (m), 1730 (m), 1683 (s), 1655 (m), 1331 (s), 1152 (s), 1104 (s), 935 (w), 812 (w); ^1H NMR ($CDCl_3$, 300 MHz) δ 7.06 (1H, d, *J* = 7.7 Hz, H-3 Ph), 6.99 (1H, dd, *J* = 7.7, 1.3 Hz, H-4 Ph), 6.98 (1H, d, *J* = 1.3 Hz, H-6 Ph), 6.08 (1H, br s, NH), 3.69 and 3.59 (1H each, each dt, *J* = 10.2, 6.2 Hz, H-6 and H'-6), 3.37 (3H, s, OCH_3), 3.22 (1H, tt, *J* = 10.8, 4.1 Hz, H-8'), 2.27 and 2.16 (3H each, each s, 2 x CH_3-Ph), 2.14 (2H, t, *J* = 7.3 Hz, H-2), 2.21–2.12 (2H, m, H-7'/H-9'), 1.90 (2H, tdd, *J* = 13.6, 7.4, 3.7 Hz, H-6'/H-10'), 1.69–1.58 (2H, m, H'-6'/H'-10'), 1.53–1.39 (4H, m, H-3 and H-5), 1.41 (9H, s, CMe_3), 1.39–1.27 (2H, m, H'-7'/H'-9'), 1.27–1.14 (2H, m, H-4); ^{13}C NMR ($CDCl_3$, 75 MHz) δ 172.9 (2C, C-1 and NCO), 172.5 (C-4'), 134.8 (C-5 Ph), 134.7 (C-1 Ph), 131.9 (C-6), 130.8 (C-2), 129.7 (C-3 Ph), 129.0 (C-4 Ph), 106.4 (C-3'), 80.2 (*C*Me_3), 77.6 (C-8'), 71.4 (C-6), 60.0 (C-5'), 55.9 (OCH_3), 35.4 (C-2), 32.7 and 32.1 (C-6' and C-10'), 29.1 (C-5), 28.59 and 28.60 (C-7' and C-9'), 28.1 (C*Me*$_3$), 25.2 (C-4), 24.7 (C-3), 21.0 and 19.7 (2 × CH_3-Ph); HRMS (TOF-MS ES+) calcd for $C_{28}H_{42}NO_5$ $[M+H]^{\cdot+}$ 472.3057, found 472.3064.

Preparation of 6-((-3-(2,5-dimethylphenyl)-8-methoxy-2-oxo-1-azaspiro[4.5]dec-3-en-4-yl)oxy)hexanoic acid (**hapten SP*h***). Treatment of *tert*-butyl ester **8** (14.5 mg, 0.031 mmol) with formic acid (0.9 mL) at room temperature for 10 h, followed by evaporation of the solvent, as previously described for the preparation of hapten SP*m*, afforded hapten SP*h* (12.7 mg, 99%).

3. Hapten activation

A solution of the hapten (1 equiv), NHS (1.1 equiv), and EDC·HCl (1.1 equiv) in anhydrous MeCN (0.3 mL per 10 µmol of hapten) was stirred at room temperature for 24 h under nitrogen atmosphere. After this time, the reaction mixture was diluted with Et_2O and washed successively with water, a 5% (w/v) aqueous solution of $NaHCO_3$, and brine. The residue obtained after evaporating the solvent was chromatographed through a short column of silica gel, using 3% (v/v) MeOH in $CHCl_3$, to give the corresponding *N*-hydroxysuccinimidyl ester.

4. Bioconjugate preparation and analysis

BSA conjugates were prepared by dropwise adding the SP*m*-NHS or SP*h*-NHS solution over a 15 mg/mL BSA solution in coupling buffer. Twenty-two moles of activated hapten SP*m* was used per mole of protein, whereas a 30-fold molar excess was employed to prepare the BSA–SP*h* conjugate. For microplate coating conjugates, the activated hapten solution (8 equiv of SP*m*-NHS or 10 equiv of SP*h*-NHS) was added over a 15 mg/mL OVA solution in coupling buffer. For enzyme tracer conjugate preparation, 6 equiv of SP*m*-NHS or 8 equiv of SP*h*-NHS (from a 5 mM activated hapten solution in DMF) was added over a 3 mg/mL HRP solution in coupling buffer. Reactions were carried out in amber glass vials by overnight incubation at room temperature with gentle stirring. Conjugates were purified using 3 serially connected 5-mL desalting columns and 100 mM phosphate buffer, pH 7.4, as eluent. The fractions containing the BSA conjugate were pooled and diluted in elution buffer, and the solution was filter sterilized with 0.45 µm sterile filters. The pool of fractions containing the OVA conjugate was diluted with elution buffer containing 0.01% (w/v) thimerosal. The enzyme tracer solutions were 1:1 (v/v) diluted in PBS containing 1% (w/v) BSA. BSA and OVA conjugates were stored frozen at −20 °C, and HRP conjugates were kept at 4 °C.

Sample preparation for MALDI-TOF analysis. A previously published procedure was followed.[5] Briefly, 100 µL of each of the protein conjugate solutions (0.5-1 mg/mL) were dialyzed against MilliQ water with Slide-A-Lyzer MINI dialysis units and then freeze-dried and lyophilized. The samples were dissolved in MilliQ H_2O to a theoretical final concentration of 1 µg/µL. Then, 1 µL of every sample solution was spotted onto the MALDI plate. After the droplets were air dried at room temperature, 1 µL of matrix (10 mg/mL sinapinic acid (Bruker) in 0.1% trifluoroacetic acid-CH_3CN/H_2O (7:3 v/v) was added and allowed to air-dry at room temperature.

Mass spectrometry analysis. A previously published procedure was followed.[5] Briefly, the resulting mixtures were analysed in a MALDI-TOF/TOF apparatus in positive linear mode (1500 shots every position) in a mass range of 10000-100000 m/z. Previously, the plate was calibrated with 1 µL of

the TOF/TOF calibration mixture (ABSciex), in 13 positions. Every sample was calibrated by 'close external calibration' method with a BSA, OVA or HRP spectrum acquired in a close position.

The final molar ratios (MR) of immunizing and assay bioconjugates determined by MALDI-TOF-MS are shown in Figures S2-S4.

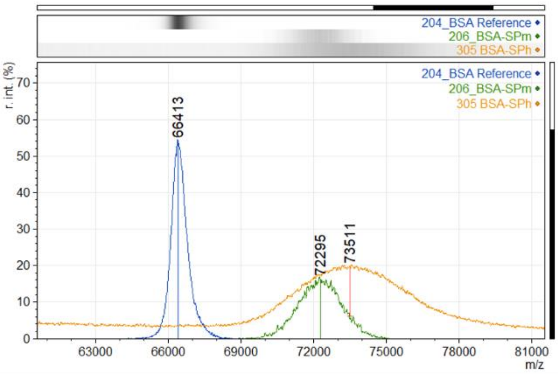

	m/z	Δm/hapten	Δ(*m/z*)	MR
**BSA**	66413			
**BSA-SP*m***	72295	455	5882	12.9
**BSA-SP*h***	73511	397	7098	17.9

Figure S2. MALDI-TOF-MS spectra of BSA (blue) and conjugates BSA-SP*m* (green) and BSA-SP*h* (orange).

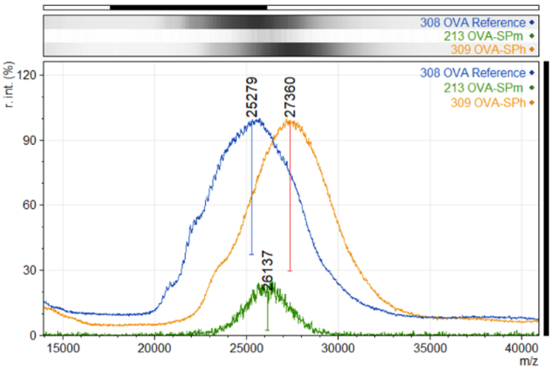

	m/z	Δm/hapten	Δ(*m/z*)	MR‡
**OVA**	25279			
**OVA-SP*m***	26137	455	858	3.8
**OVA-SP*h***	27360	397	2081	10.5

‡ Based on doubly charged protein and conjugate ions

Figure S3. MALDI-TOF-MS spectra of OVA (blue) and conjugates OVA-SP*m* (green) and OVA-SP*h* (orange).

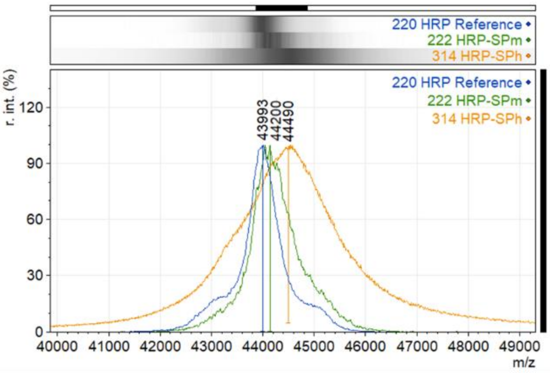

	m/z	Δm/hapten	Δ(*m/z*)	MR
**HRP**	43993			
**HRP-SP*m***	44200	455	207	0.5
**HRP-SP*h***	44490	397	459	1.2

Figure S4. MALDI-TOF-MS spectra of HRP (blue) and conjugates HRP-SP*m* (green) and HRP-SP*h* (orange).

5. Antibody generation and selection

Mouse immunization. Each mouse received, every three weeks, 100 µg of conjugate in a 1:1 (v/v) emulsion (200 µL) between 100 mM phosphate buffer, pH 7.4, and Freund's adjuvant (complete for the first injection and incomplete for the second and third injections). After a resting period of at least three weeks from the third boost, a fourth injection was applied four days before cell fusion, with 100 µg of conjugate in sterile phosphate buffer.

Hybridoma generation. A previously published procedure was followed.[6] Briefly, hybridoma cells were generated by fusion between lymphocyte cells from two equally immunized mice and mouse myeloma cells. Cell fusion was performed at a 4:1 ratio using 1 mL of PEG 1500 as the fusing agent. Fused cells were distributed in 96-well culture plates at a density of 1.5×10^5 lymphocytes per well in 100 µL of DMEM containing 15% (v/v) FBS. Twenty-four hours after plating, 100 µL of selection medium (DMEM supplemented with HAT) containing 20% (v/v) FBS and 1% (v/v) HFCS was added to each well.

Hybridoma screening. Hybridoma culture supernatants were assayed, twelve days after cell fusion, following a sequential double-screening process as previously described in Mercader et al.[6] First, cell cultures were screened by indirect differential cELISA with plates coated at 0.1 mg/mL with homologous OVA conjugate. Each culture supernatant was analysed in parallel with and without spirotetramat – 50 µL of cell culture supernatant was added to two adjacent wells of a coated ELISA plate, one containing 50 µL of PBS (blank) and the other containing 50 µL of 100 nM spirotetramat in PBS – and the obtained assay signals were compared. The immunochemical reaction was detected with RAM-HRP diluted 1/2000 in PBS-T. The retained peroxidase activity was revealed as described in the manuscript using *o*-phenylendiamine and stopped with 1 M H_2SO_4. The absorbance was read at 492 nm. In a second screening assay, culture supernatants from those wells that contained antibodies with interesting binding properties, according to the first screening experiment, were re-evaluated by checkerboard indirect cELISA. Serial dilutions of the supernatant were assayed with different spirotetramat or spirotetramat-enol concentrations (0, 10, and 100 nM) using ELISA plates coated with antigen conjugate at 0.1 µg/mL. Selected hybridomas were cloned by limiting dilution in cloning medium (DMEM containing 20% (v/v) FBS and supplemented with HT and 1% (v/v) HFCS). Stable antibody-producing clones were expanded and cryopreserved in liquid nitrogen.

---

[6] J. V. Mercader, C. Suárez-Pantaleón, C. Agulló, A. Abad-Somovilla, A. Abad-Fuentes. Production and characterization of monoclonal antibodies specific to the strobilurin pesticide pyraclostrobin. *J. Agric. Food Chem.* **56**, 7682–7690 (2008).

6. Antibody specificity

Table S1. Specificity of the selected mAb (n=3).

Analyte	CR (%)
SP	11.8
SP-enol	100
SP-keto	0.02
SP-mono	0.19
SP-glu	5.11

7. Direct competitive ELISA procedure

The same protocol previously described for this format was applied.[7] Briefly, plates were coated with 100 µL per well of 1 µg/mL GAM solution in carbonate–bicarbonate buffer, pH 9.6, by overnight incubation at 4 °C. Wells were washed four times with washing solution (150 mM NaCl with 0.05% (v/v) Tween-20) after each incubation step. Then, 100 µL per well of specific mAb solution in PBS-T was added and plates were incubated at room temperature during 1 h. The competitive reaction was carried out by adding 50 µL per well of analyte solution or diluted sample and 50 µL per well of tracer solution. The retained peroxidase activity was revealed with 100 µL per well of freshly prepared enzyme substrate solution (2 mg/mL *o*-phenylendiamine and 0.012% (v/v) H_2O_2 in 25 mM sodium citrate and 62 mM sodium phosphate buffer, pH 5.4). Ten minutes later, the enzyme reaction was stopped by adding 100 µL per well of 1 M H_2SO_4.

8. Influence of pH and ionic strength

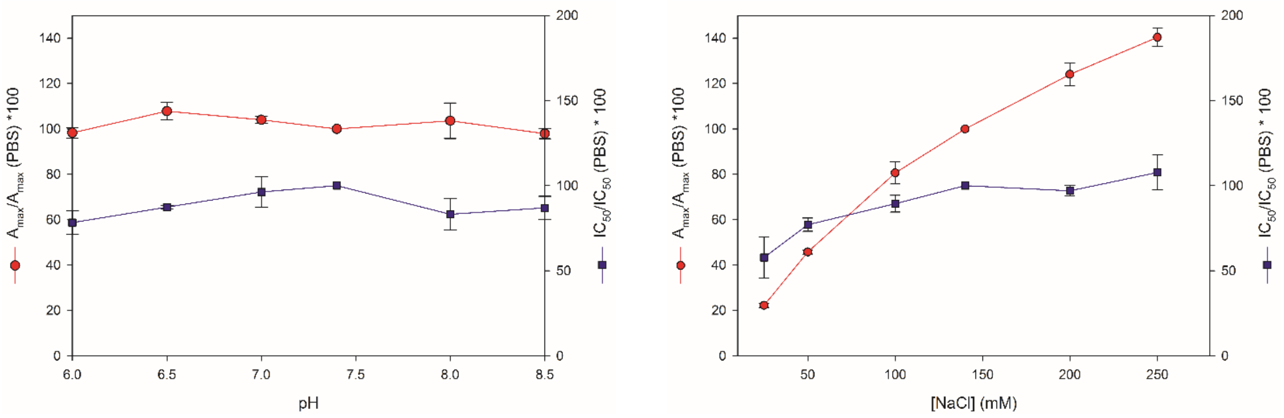

Figure S5. Influence of pH and ionic strength over the A_{max} and IC_{50} values of the spirotetramat-enol standard curve of the studied direct cELISA.

[7] E. Ceballos-Alcantarilla, D. López-Puertollano, C. Agulló, A. Abad-Fuentes, A. Abad-Somovilla, J. V. Mercader. Combined heterologies for monoclonal antibody-based immunoanalysis of fluxapyroxad. *Analyst* **143**, 5718–5727 (2018).

9. Influence of ethanol and acetonitrile

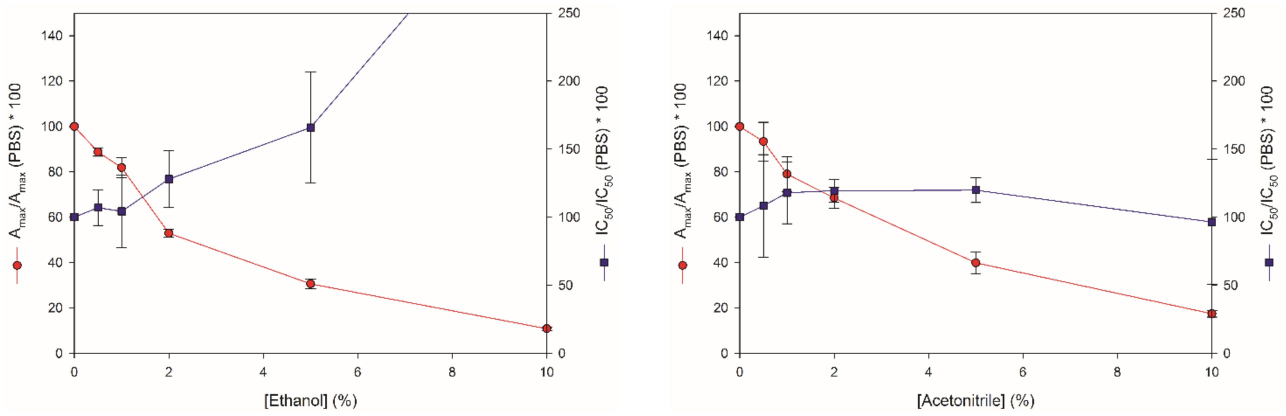

Figure S6. Influence of ethanol and acetonitrile over the A_{max} and IC_{50} values of the spirotetramat-enol standard curve of the studied direct cELISA.

10. Matrix effects

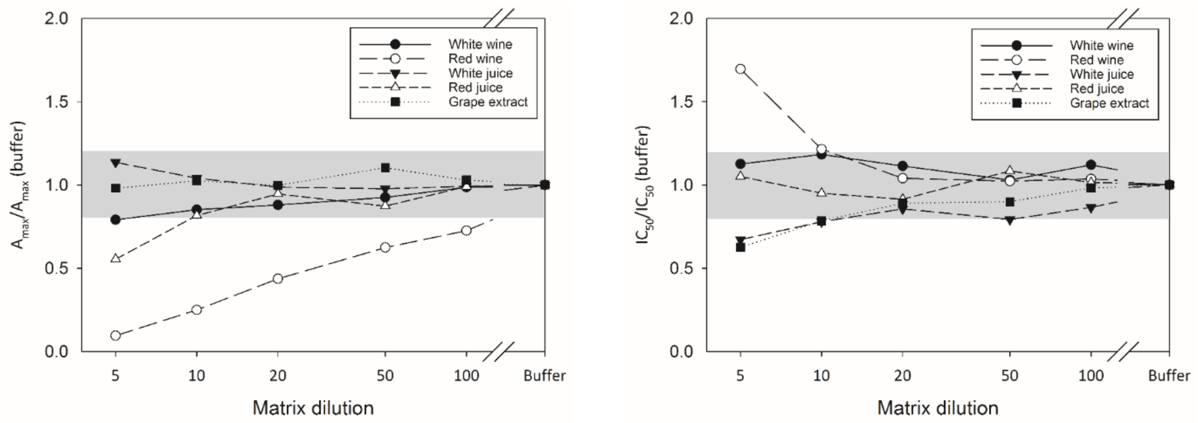

Figure S7. Influence of wine, grape juice and grape extracts over the A_{max} and IC_{50} values of the spirotetramat-enol standard curve of the studied direct cELISA. Every standard solution was subjected to a hydrolysis step before assaying. The grey bar indicates the ±20% deviation range from the A_{max} and IC_{50} value obtained in buffer.

11. Immunoassay validation by method comparison

Table S2. Analysis of extracts from grapes sprayed with Movento Gold.

Sample	HPLC–MS[a] (ng/mL)	Direct cELISA[b] (ng/mL)	Bias (%)
M0	8.6 ± 0.8	9.6 ± 6.5	111.6
M1	441.8 ± 27.3	400.8 ± 28.2	90.7
M2	299.4 ± 12.2	251.0 ± 17.0	83.8
M3	138.9 ± 6.2	106.4 ± 4.0	76.6
M4	1205.6 ± 87.9	1187.2 ± 55.7	98.5
M5	712.0 ± 43.0	732.5 ± 54.8	102.9
M6	216.4 ± 3.7	187.7 ± 11.5	86.7
M7	41.4 ± 1.3	37.8 ± 2.6	91.3
M8	28.9 ± 1.0	24.3 ± 1.0	84.1
M9	15.1 ± 0.7	11.3 ± 0.6	74.8
M10	128.2 ± 1.4	109.7 ± 1.1	85.6
M11	69.8 ± 0.5	66.5 ± 3.6	95.3
M12	21.4 ± 0.5	18.0 ± 0.7	84.1

[a] Results (n=3) are the sum of spirotetramat and spirotetramat-enol concentration. The contents of other metabolites were negligible.
[b] Absorbance results (n=5) were interpolated from a spirotetramat-enol standard curve.

12. Optimization of the lateral flow immunoassay

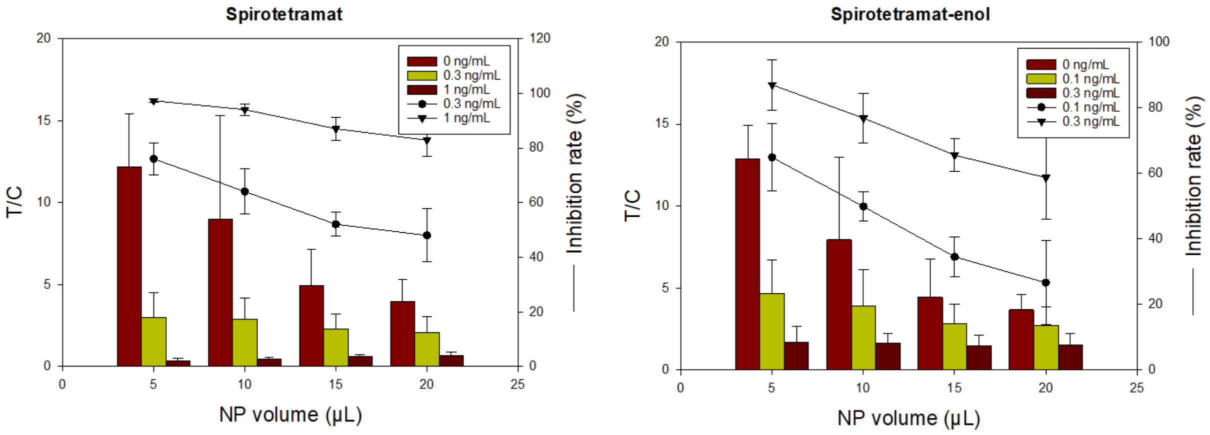

Figure S8. Optimization of the NP volume.

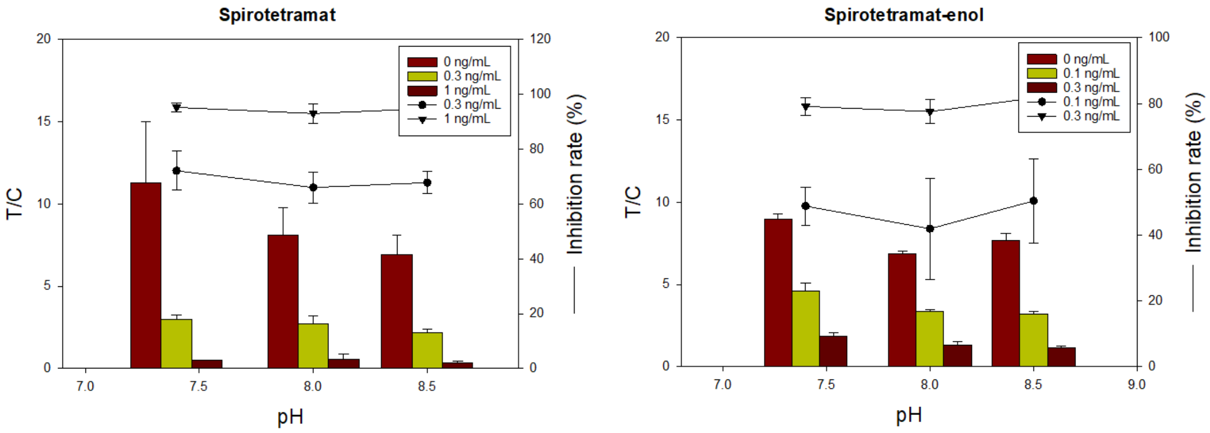

Figure S9. Optimization of pH.

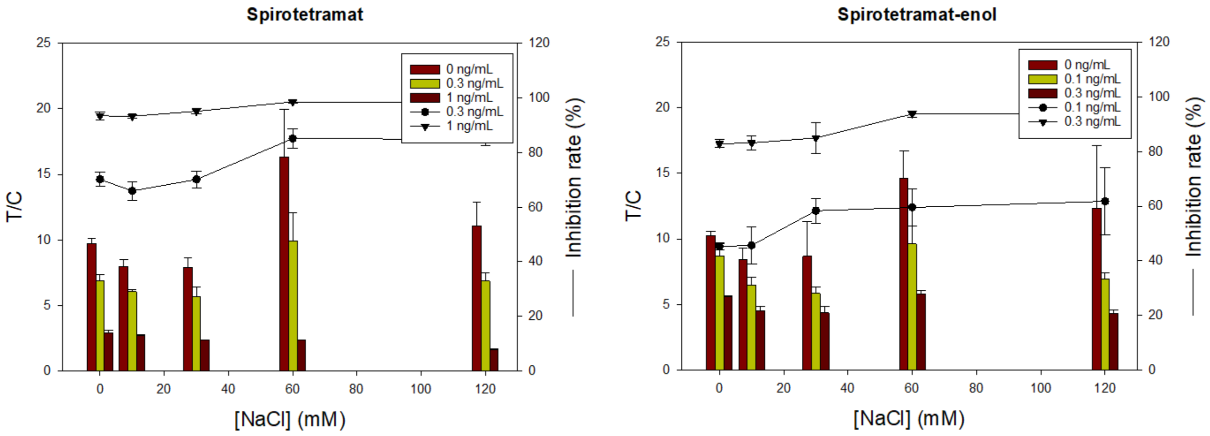

Figure S10. Optimization of the ionic strength.

13. Validation of immunochromatographic tests

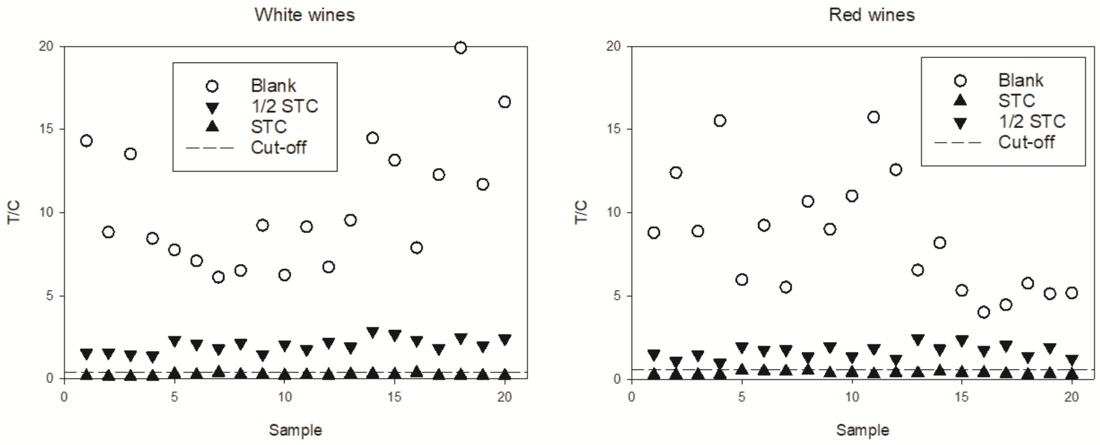

Figure S11. Validation of the developed lateral flow immunoassay using SP-enol fortified wine samples.

Table S3. Validation of the lateral flow immunoassay to determine SP-enol in wine samples (n = 20).

	White wines		Red wines	
	STC[a]	1/2 STC	STC	1/2 STC
Average T/C	0.2	2.0	0.4	1.7
CV (%)	27.3	20.7	25.7	24.4
Cut-off (5% negative results)	0.4	2.8	0.5	2.4
False suspect (%) from blank	0.8	3.0	0.3	1.8
False suspect (%) from 1/2 STC	0.04	-	0.5	-
False negative (%) from STC	-	0.00	-	0.00

[a] The STC was fixed at 1000 ng/mL.

14. ^{1}H NMR spectra of haptens SP*m* and SP*h*

^{1}H NMR spectrum of hapten SP*m* ($CDCl_3$, 300 MHz)

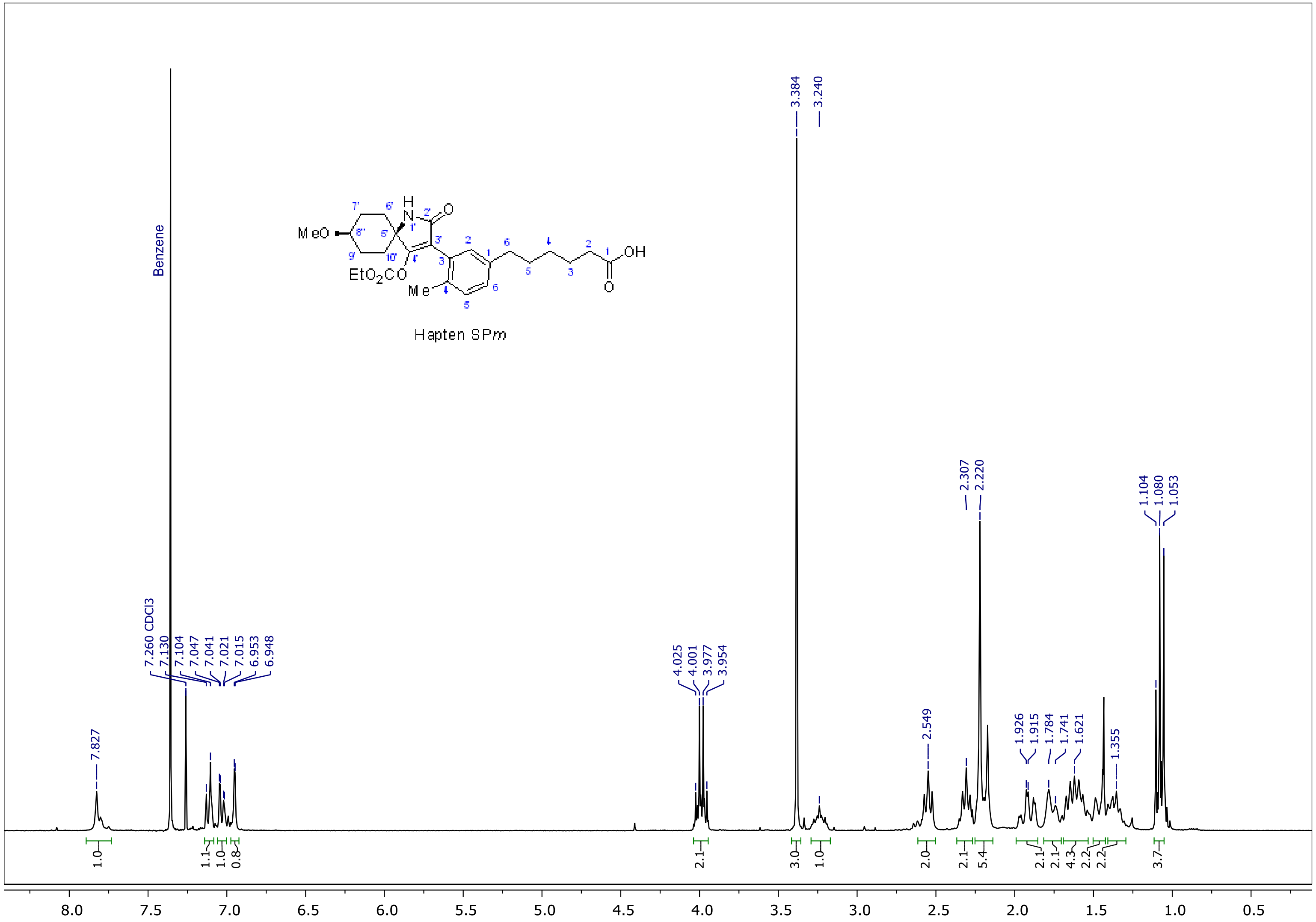

^{1}H NMR spectrum of hapten SP*h* ($CDCl_3$, 300 MHz)

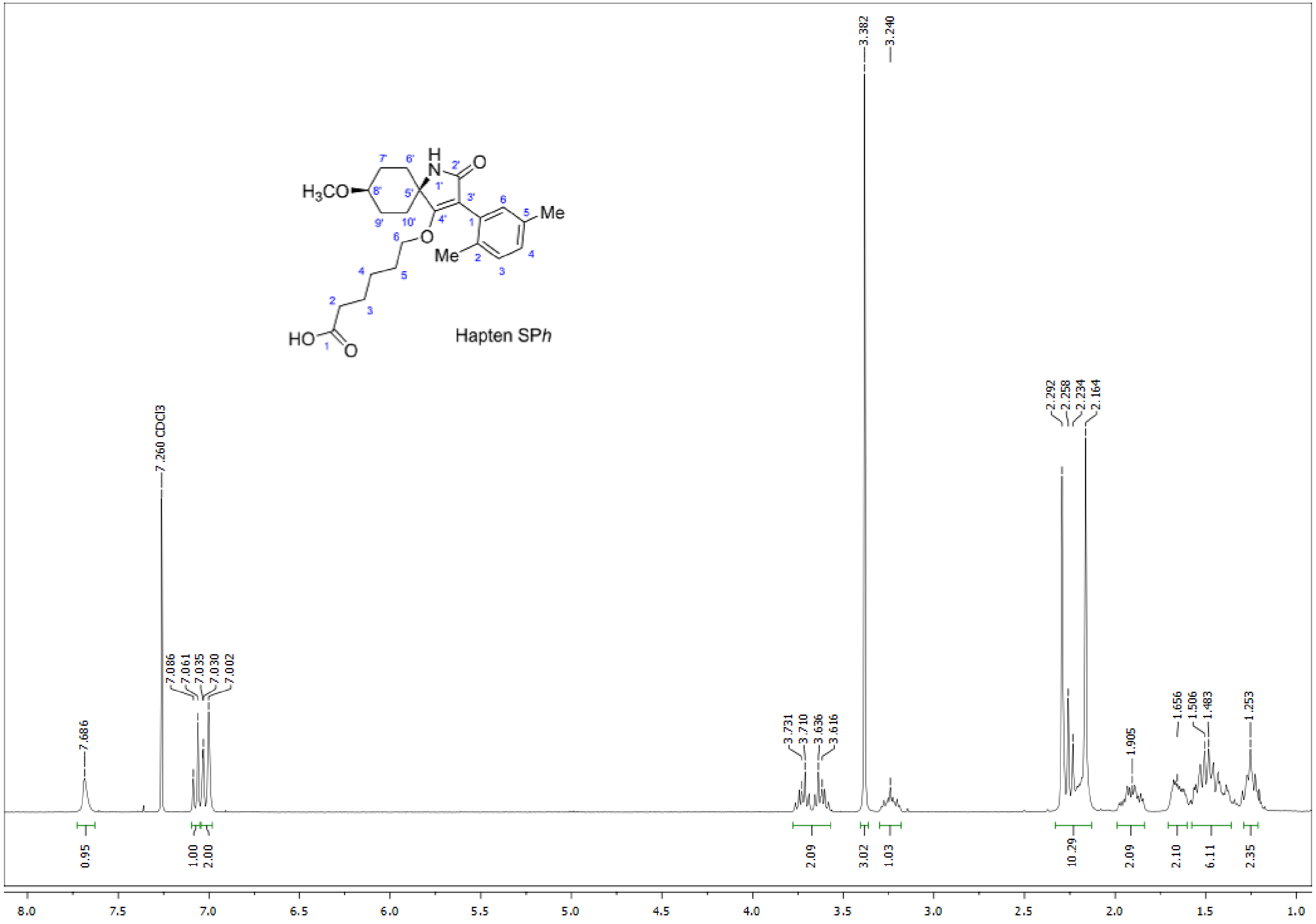

Supplement: Supplementary file 1 — Supplementary Information. [file 41598_2021_81432_MOESM1_ESM.pdf]
